# The proofreading exonuclease of leading-strand DNA polymerase epsilon prevents replication fork collapse at broken template strands

**DOI:** 10.1093/nar/gkad999

**Published:** 2023-11-08

**Authors:** Tasnim Ahmad, Ryotaro Kawasumi, Tomoya Taniguchi, Takuya Abe, Kazuhiro Terada, Masataka Tsuda, Naoto Shimizu, Toshiki Tsurimoto, Shunichi Takeda, Kouji Hirota

**Affiliations:** Department of Chemistry, Graduate School of Science, Tokyo Metropolitan University, Minamiosawa 1-1, Hachioji-shi, Tokyo 192-0397, Japan; Department of Chemistry, Graduate School of Science, Tokyo Metropolitan University, Minamiosawa 1-1, Hachioji-shi, Tokyo 192-0397, Japan; Department of Chemistry, Graduate School of Science, Tokyo Metropolitan University, Minamiosawa 1-1, Hachioji-shi, Tokyo 192-0397, Japan; Department of Chemistry, Graduate School of Science, Tokyo Metropolitan University, Minamiosawa 1-1, Hachioji-shi, Tokyo 192-0397, Japan; Department of Radiation Genetics, Graduate School of Medicine, Kyoto University, Yoshidakonoe, Sakyo-ku, Kyoto 606-8501, Japan; Department of Radiation Genetics, Graduate School of Medicine, Kyoto University, Yoshidakonoe, Sakyo-ku, Kyoto 606-8501, Japan; Program of Mathematical and Life Sciences, Graduate School of Integrated Sciences for Life, Hiroshima University, 1-3-1, Kagamiyama, Higashi-Hiroshima, Hiroshima 739-8526, Japan; Division of Genetics and Mutagenesis, National Institute of Health Sciences, 3-25-26 Tonomachi, Kawasaki-ku, Kawasaki 210-9501, Japan; Department of Radiation Genetics, Graduate School of Medicine, Kyoto University, Yoshidakonoe, Sakyo-ku, Kyoto 606-8501, Japan; Program of Mathematical and Life Sciences, Graduate School of Integrated Sciences for Life, Hiroshima University, 1-3-1, Kagamiyama, Higashi-Hiroshima, Hiroshima 739-8526, Japan; Department of Biology, Faculty of Science, Kyushu University, 744 Motooka, Nishi-ku, Fukuoka 819-0395, Japan; Shenzhen University, School of Medicine, Shenzhen, Guangdong 518060, China; Department of Chemistry, Graduate School of Science, Tokyo Metropolitan University, Minamiosawa 1-1, Hachioji-shi, Tokyo 192-0397, Japan

## Abstract

Leading-strand DNA replication by polymerase epsilon (Polϵ) across single-strand breaks (SSBs) causes single-ended double-strand breaks (seDSBs), which are repaired via homology-directed repair (HDR) and suppressed by fork reversal (FR). Although previous studies identified many molecules required for hydroxyurea-induced FR, FR at seDSBs is poorly understood. Here, we identified molecules that specifically mediate FR at seDSBs. Because FR at seDSBs requires poly(ADP ribose)polymerase 1 (PARP1), we hypothesized that seDSB/FR-associated molecules would increase tolerance to camptothecin (CPT) but not the PARP inhibitor olaparib, even though both anti-cancer agents generate seDSBs. Indeed, we uncovered that Polϵ exonuclease and CTF18, a Polϵ cofactor, increased tolerance to CPT but not olaparib. To explore potential functional interactions between Polϵ exonuclease, CTF18, and PARP1, we created exonuclease-deficient *POLE1^exo^*^−/−^, *CTF18^−/−^*, *PARP1^−/−^*, *CTF18*^−/−^/*POLE1^exo^*^−/−^, *PARP1*^−/−^/*POLE1^exo^*^−/−^, and *CTF18*^−/−^/*PARP1^−/−^* cells. Epistasis analysis indicated that Polϵ exonuclease and CTF18 were interdependent and required PARP1 for CPT tolerance. Remarkably, *POLE1^exo−/−^* and HDR-deficient *BRCA1^−/−^* cells exhibited similar CPT sensitivity. Moreover, combining *POLE1^exo−/−^* with *BRCA1^−/−^* mutations synergistically increased CPT sensitivity. In conclusion, the newly identified PARP1-CTF18-Polϵ exonuclease axis and HDR act independently to prevent fork collapse at seDSBs. Olaparib inhibits this axis, explaining the pronounced cytotoxic effects of olaparib on HDR-deficient cells.

## Graphical Abstract

**Graphical Abstract:**
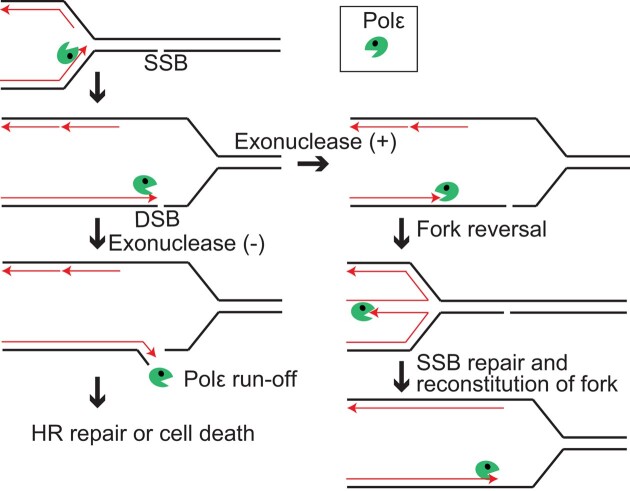

## Introduction

An estimated 55000 single-strand breaks (SSBs) occur daily in the DNA of individual cells (1). The induction of excessive SSBs is a common mechanism by which malignant cells are destroyed (2,3). SSBs on leading-strand replication template strands can generate double-strand breaks (DSBs), termed single-ended DSBs (seDSBs) (Supplementary Figure S1, step 1), via replication run-off, leading to replication fork collapse (4–6). Repair of seDSBs is mediated via various mechanisms in eukaryotic cells (reviewed in (7), including homology-directed repair (HDR) (8,9) (Supplementary Figure S1, steps 3 to 5), a process in which BRCA1 has a pivotal role (10,11). HDR defects cause hypersensitivity to the anti-cancer agents camptothecin (CPT), olaparib (a PARP1 inhibitor, PARPi), and cisplatin (a DNA crosslinker) (12).

Cells can also prevent seDSB formation (42) (reviewed in (7) through mechanisms that depend on reversing replication fork progression before SSBs (Supplementary Figure S1, step 2) (13,14); this reversal is associated with fork slowing. Previous studies extensively characterized the fork reversal (FR) mechanism by inhibiting replicative DNA polymerases through extended exposure to hydroxyurea (13,14). As hydroxyurea simultaneously inhibits the progression of all forks (reviewed in (7), the fate of stalled forks, including FR, can be accurately examined using the DNA fiber assay. Nonetheless, the mechanisms identified in the context of hydroxyurea exposure may be irrelevant to the process of FR that occurs during exposure to CPT, which induces SSBs. Importantly, CPT does not simultaneously block the progression of all forks; instead, the timing of the arrest of individual replication forks differs. The interpretation of DNA fiber assay data in this context is, therefore, less straightforward (15–20), and the molecular mechanisms underlying FR at broken template strands remain poorly characterized. To date, PARP1 and RECQ1 are among the few factors confirmed to be involved in FR at broken template strands. PARP1 promotes FR, while RECQ1 antagonizes PARP1 by resolving reversed forks (15,18,21–23). Although the maintenance of hydroxyurea-induced FR requires HDR factors (2,13,14), the functional relationship between HDR and FR at seDSBs remains unclear. Previous studies examined FR at seDSBs by inducing excessive SSBs using olaparib or CPT, a topoisomerase 1 (TOP1) inhibitor widely used in anti-cancer therapy (2,3,18,21,24,25). The synthetic lethality of PARPi in HDR-deficient cells provides a novel therapeutic strategy to cure patients with HDR-deficient cancers (2,26). There are currently four PARPi in clinical use for the treatment of several types of cancer; however, most patients acquire PARPi resistance following the prolonged administration of these drugs (2,26). Previous studies have not fully elucidated the molecular mechanisms underlying PARPi resistance, and the role of PARP1-dependent FR in PARPi resistance remains poorly understood (2,26).

Both PARPi and CPT kill cancer cells by inducing seDSBs. During the TOP1-mediated cycle of DNA nicking and resealing (25,27), CPT blocks the resealing step. This activity induces the formation of SSBs covalently associated with TOP1 at their 3′ ends, termed stalled TOP1ccs (Supplementary Figure S1, top) (25,27). When DNA polymerase epsilon (Polϵ) encounters stalled TOP1ccs during leading-strand replication, these SSBs are converted to seDSBs (4–6) (Supplementary Figure S1, step 1). Recent CRISPR-Cas9 screens against genotoxic agents indicated that the lethal dose of CPT for PARP1-deficient retinal pigment epithelium (RPE) cells was approximately 1000 times lower than that for wild-type cells (12) (Supplementary Figure S2A), demonstrating that PARP1 loss dramatically increases CPT sensitivity. This marked hypersensitivity is attributable to two mechanisms: [1] PARP1-promoted tyrosyl-DNA phosphodiesterase 1 (TDP1)-dependent removal of 3′ TOP1 adducts from stalled TOP1ccs (27–30), and [2] PARP1-promoted FR at stalled TOP1ccs on template strands (18,21,24,25) (Supplementary Figure S1, step 2). Previous studies have not yet defined the contribution of the second mechanism to cellular tolerance to CPT because of the lack of known factors functioning specifically in this process.

Polϵ, consisting of four subunits (PolE1, PolE2, PolE3 and PolE4), functions as a replicative polymerase in leading-strand synthesis (31,32). The PolE1 subunit possesses polymerase and proofreading 3′–5′ exonuclease activities (33–37). The latter activity (hereafter termed Polϵ exonuclease activity) eliminates misincorporated deoxynucleotides, ribonucleotides, and nucleoside analogs (38–42). Although this proofreading activity is the only known function of Polϵ exonuclease (42–44), biochemical studies have documented robust 3′–5′ exonucleolytic activity that efficiently digests nascent DNA strands even in the presence of physiological concentrations of deoxynucleotides (38,45). A genetic study in yeast also demonstrated the robust exonucleolytic activity of stalled Polϵ *in vivo* (46). Nonetheless, it remains unclear whether this vigorous activity has any physiological function in genome maintenance.

In this study, we aimed to investigate the role of the proofreading exonucleolytic activity of Polϵ in genome maintenance. As our initial assays showed that Polϵ-exonuclease-deficient cells exhibited hypersensitivity to CPT but not to olaparib, we hypothesized that Polϵ exonuclease activity might contribute to CPT tolerance via PARP1-dependent FR at seDSBs (Supplementary Figure S1, step 2) (reviewed in (2,7). In addition, to identify further factors required for this process, we performed data mining on published sensitivity data to CPT and olaparib (12). This analysis showed that CTF18, a component of the leading-strand replisome (47,48), contributed to cellular tolerance to CPT in collaboration with Polϵ exonuclease. We therefore went on to investigate the epistatic relationship between CTF18, Polϵ, and PARP1 by generating *POLE1^exo^*^−/−^, *CTF18^−/−^*, *PARP1^−/−^*, *CTF18*^−/−^/*POLE1^exo^*^−/−^, *PARP1*^−/−^/*POLE1^exo^*^−/−^ and *CTF18*^−/−^/*PARP1^−/−^* cells. These genetic analyses revealed the PARP1-Polϵ exonuclease-CTF18 axis as a novel leading-strand replication-protecting pathway that counteracts seDSB formation, most likely via PARP1-dependent FR (Supplementary Figure S1, step 2). Further genetic analyses elucidated that the PARP1-Polϵ exonuclease-CTF18 axis increased CPT tolerance independently of TDP1-mediated TOP1ccs repair and HDR. Together, our findings highlight the previously unappreciated role of Polϵ exonuclease in replication fork protection at broken templates and may provide important clues to improving cancer chemotherapy.

## Materials and methods

### DT40 and TK6 cell cultures

The DT40 cell line (49) was cultured in D-MEM/Ham's F-12 medium supplemented with 10 μM β-mercaptoethanol, penicillin (100 U/ml), streptomycin (100 μg/ml), l-glutamine (2 mM), 10% fetal calf serum, and 1% chicken serum (Thermo Fisher Scientific Inc., Waltham, MA, USA) at 39.5°C. TK6 cells (50) were obtained from the JCRB Cell Bank (https://cellbank.nibiohn.go.jp/). These cells were cultured in Roswell Park Memorial Institute (RPMI) 1640 medium (Nacalai Tesque, Kyoto, Japan) supplemented with 10% heat-inactivated horse serum (Thermo Fisher Scientific Inc., Waltham, MA, USA) and sodium pyruvate (0.1 mM), L-glutamine (2 mM), penicillin (100 U/ml), and streptomycin (100 μg/ml) (Nacalai Tesque, Kyoto, Japan) at 37°C.

### Chicken DT40 and human TK6 strains and genotoxic reagents

Previously established *POLE1^exo^*^−/−^ (38) and *PARP1*^−/−^ (51,52) cells generated from DT40 and TK6 cells, and previously established *BRCA1*^−/−^ (53), *TDP1*^−/−^ (28), and *CTF18*^−/−^ (54) DT40 cells were used. *RECQ1* was disrupted in DT40 cells as previously described (55). The DT40 and TK6 cell lines used are listed in Supplementary Table S1. Camptothecin (CTP) (Topogen, CO, USA) and olaparib (Funakoshi, Tokyo, Japan) were used for sensitivity assays; these drugs were dissolved in dimethyl sulfoxide.

### Generation of *POLE1^exo−/−^/AAVS1::ef-1α pro-POLE1 cDNA* cells

Human *POLE1* cDNA was amplified from a cDNA library (using primers 5′-ATGTCTCTGAGGAGCGGCGG-3′ and 5′-CTAATGGCCCAGCTGTGGGTTC-3′) and inserted downstream of the *ef-1α* promoter in the *AAVS1* targeting vector carrying the blasticidin-S resistance gene. The CRISPR expression vector for the CRISPR-Cas9 system was designed to digest the *AAVS1* targeting region as previously described (56). Integration of the *POLE1* cDNA expression system into the *AAVS1* locus was confirmed by PCR using primers 5′-CTTCTCTGTCGCTACTTCTACTAATTCTAG-3′ and 5′-GTTGGAGGAGAATCCACCCAAAAGGCAG-3′.

### Measurement of cellular sensitivity to DNA-damaging agents

To measure cellular sensitivity to CPT, a liquid-culture cell survival assay was performed as previously described (57,58). Briefly, DT40 or TK6 cells were diluted in medium (0.5 × 10^4^ cells/ml) and dispensed into 24-well plates (1 ml per well). CPT was then added and mixed, and the cells were cultured for 48 h (DT40) or 72 h (TK6) before being transferred to 96-well plates (100 μl per well). ATP levels were measured using the CellTiter-Glo Cell Viability Assay (Promega, MA, USA) according to the manufacturer's instructions. Luminescence was measured using a Fluoroskan Ascent FL Microplate Fluorometer and Luminometer (Thermo Fisher Scientific Inc., Waltham, MA, USA).

### Chromosomal aberration analysis

Mitotic chromosome spreads were prepared and analyzed as previously described (59). DT40 or TK6 cells were arrested in the M phase by treatment with colcemid (Thermo Fisher Scientific Inc., Waltham, MA, USA; 0.1 μg/ml) for 3 h. The cells were pelleted by centrifugation (1200 rpm for 5 min), resuspended in 75 mM KCl (10 ml) for 13 min at room temperature, and fixed in a freshly prepared 3:1 mixture of methanol and acetic acid (Carnoy's solution; 2 ml). The pelleted cells were then resuspended in Carnoy's solution (7 ml), dropped onto cold glass slides (approximately 10^6^ cells per slide), and air-dried. The slides were stained in 5% HARLECO Giemsa Stain solution (Nacalai Tesque) for 10 min, rinsed with water and acetone, and dried at 20°C. The slides were examined under a microscope (ECLIPSE-Ni, NIKON, Tokyo, Japan) at 1000× magnification, and the chromosomes in each mitotic cell were scored.

### Measurement of sister chromatid exchanges

Sister chromatid exchanges (SCEs) were measured as previously described, with slight modifications (60). Briefly, DT40 cells were incubated in a medium containing CPT (2 nM) and 5-bromo-2′-deoxy-uridine (BrdU) (10 μM) at 39.5°C for 16 h, corresponding to two cell cycle periods for these cells. TK6 cells were incubated with CPT (0.5 nM) and BrdU (10 μM) for 24 h at 37°C. Both cell lines cells were treated with colcemid (0.1 μg/ml) for the last 2.5 h of the incubation to enrich mitotic cells. The cells were then pelleted by centrifugation (1200 rpm for 5 min), resuspended in 75 mM KCl (0.2 ml) for 13 min at 20°C, and fixed in freshly prepared Carnoy's solution (10 ml). The pelleted cells were resuspended in Carnoy's solution (0.4 ml), dropped onto clean glass slides (Matsunami glass, Osaka, Japan), and air-dried. The dried slides were incubated with Hoechst 33258 nuclei acid stain (10 μg/ml) in phosphate buffer (pH 6.8) for 20 min and rinsed with McIlvaine buffer (164 mM Na_2_HPO_4_ and 16 mM citric acid pH 7.0). Next, the slides were irradiated using black light (λ = 352 nm) for 25 min and incubated in saline-sodium citrate (0.15 M NaCl plus 0.015 M sodium citrate) solution at 58°C for 20 min, after which they were stained with 5% HARLECO Giemsa Stain Solution (Nacalai Tesque, Kyoto, Japan) for 10 min. The slides were examined under a microscope (ECLIPSE-Ni, NIKON, Tokyo, Japan) at 1000 × magnification, and all Giemsa-stained metaphase cells were scored per test. Standard error was calculated as the square root of the number of breaks based on the Poisson distribution of chromosomal aberrations (61).

### Immunofluorescent visualization of subnuclear RAD51 foci

The experimental conditions for immunocytochemical analysis were previously described (62). Briefly, following treatment of the indicated DT40 cells with CPT for 16 h at 39.5°C, the cells were collected on glass slides using a Cytospin apparatus (Shandon, Pittsburgh, PA, USA). The cells were fixed with 4% formaldehyde for 10 min at room temperature, permeabilized with 0.5% TritonX-100, and, after two rinses in phosphate-buffered saline (PBS), were blocked using PBS/3% bovine serum albumin (BSA). The cells were then incubated with an anti-RAD51 antibody (Bio Academia, Osaka, Japan, clone 70–005, diluted 1/500) in PBS/3% BSA for 1 h at room temperature. After three washes in PBS, the cells were incubated with Alexa Fluor 488 goat anti-rabbit IgG antibody (Invitrogen, diluted 1/1000) in PBS/5% BSA for 1 h at room temperature. After three rinses in PBS times, the cells were counterstained with 4′,6′-diamidino-2-phenylindole (DAPI) and examined under a microscope (ECLIPSE-Ni, NIKON, Tokyo, Japan). At least 100 cells were scored per data point.

### DNA fiber assays

DNA fiber assays were performed as previously described (63,64), slightly modifying the labeling method used for the replication tracts. Briefly, cells were sequentially labeled for 15 min (for DT40) or 20 min (for TK6), each with 25 μM 5-chloro-2′-deoxyuridine (CldU) and 250 μM 5-iodo-2′-deoxyuridine (IdU). Fiber length was measured using ImageJ software (https://imagej.nih.gov/ij/docs/faqs.html), and the CldU/IdU ratio was calculated. Measurements were recorded from areas of the slides with untangled DNA fibers to prevent the possibility of recording labeled patches from tangled bundles of fibers.

### Pulsed-field gel electrophoresis (PFGE) detection of DSBs

Genomic DNA was gently purified from an agarose plug and analyzed by PFGE (Bio-Rad, CA, USA), as described previously (65). A previous study (65) showed the PFGE of the *S.cerevisiae* genome (the largest available MW marker) along with genomic DNA derived from non-irradiated and IR-treated human cells. The PFGE showed a single stacked band with the genomic DNA of IR-irradiated cells. This genomic DNA migrated in the PFGE in the same manner as *S. cerevisiae* genome containing 16 intact chromosomes (0.225–2.2 Mb), which also showed a single stacked band. However, intact human genomic DNA (over 50 Mbp per chromosome) did not enter PFGE gel. Thus, a single stacked band contains fragmented DNAs of 0.225–2.2 Mb but not intact chromosomes (over 50 Mb).

### Neutral comet assay

DT40 cells (5 × 10^4^) were suspended in 0.75% low melting point agarose (made up in PBS) and dropped onto slides pre-coated in 1% agarose. The mounted cells were lysed by incubating in lysis buffer (2.5 M NaCl, 100 mM EDTA, 10 mM Tris–HCl pH 10, 0.5% Triton-X, 1.0% *N*-lauroylsarcosine sodium salt) for 2 h and run in electrophoresis buffer (0.3 M sodium acetate, 100 mM Tris–HCl pH 8.3) at 25 V for 1 h, at 4°C. After electrophoresis, the slides were washed with 1× PBS and dehydrated in 100% ethanol for 10 min. The slides were then dried at 37°C for 30 min and stained with 1 × SybrGold solution (Invitrogen, CA, USA). Images were captured using a BZ-X810 fluorescence microscope (Keyence, Tokyo, Japan). Tail DNA percentage, reflecting the number of DSBs, was measured for cells exposed to 0 or 20 nM CPT for 1 h. OpenComet (66) was used to quantify the tail DNA percentage;150 cells were scored per sample.

### Statistical analysis

*t*-tests were used to test for significant differences in cellular survival, DNA fiber length, and number of chromosome breaks. The Mann—Whitney–Wilcoxon test was used for statistical analyses of neutral comet assays and symmetricity analysis of DNA fibers.

## Results

### Exonuclease-deficient Polϵ cells exhibit hypersensitivity to CPT but not olaparib

**Figure 1.**
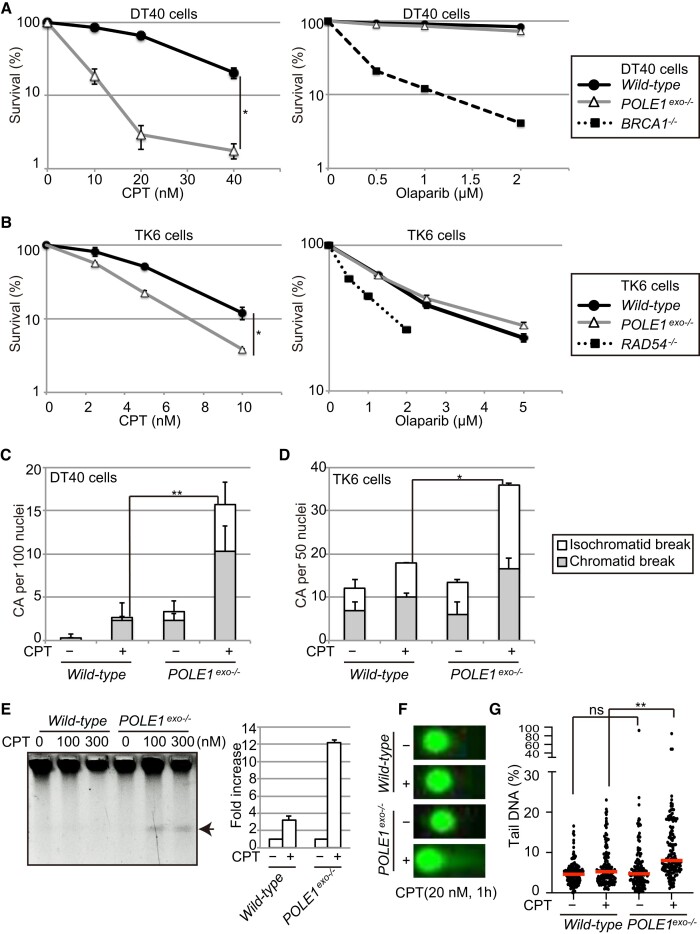
The proofreading exonuclease activity of Polϵ is involved in the suppression of DSBs in cells treated with CPT. (A, B) Sensitivity of DT40 (**A**) and TK6 (**B**) cells with the indicated genotypes to camptothecin (CPT) or olaparib treatment for 48 and 72 h, respectively. Data represent means ± standard deviation (SD) from two independent experiments; Student's *t*-test *P< 0.05. (C, D) Polϵ proofreading exonuclease activity prevents CPT-induced chromosomal breakage. DT40 (**C**) and TK6 cells (**D**) with the indicated genotypes were cultured with 10 nM CPT for 16 h or 5 nM CPT for 12 h, respectively, and colcemid was added to the cultures for the last 3 h. The numbers of chromosomal aberrations (CAs) before and after CPT treatment were scored. Data represent means ± standard deviation (SD) from three (DT40) or two (TK6) independent experiments. (Data from individual experiments are shown in Supplementary Figure S3A.) Breaks in one of the sister chromatids are categorized as chromatid-type breaks. Breaks at the same site of the two sister chromatids are categorized as isochromatid-type breaks. (**E**) Representative image showing pulsed-field gel electrophoresis analysis of DSBs induced in cells by treatment with CPT (0, 100 or 300 nM) for 1 h. Quantifications of DSBs. The intensity of the DSB fractions was quantified and presented as the fold increase of the DSB induced by CPT (300 nM). Data represent means and standard deviation (SD) from two independent experiments. (F, G) Neutral comet analysis with/without exposure to CPT (20 nM) for 1 h. Representative images are shown in (**F**); individual dots in (**G**) show the percentages of the tail fraction in the analyzed nuclei. At least 150 nuclei were scored per analysis. Red bars represent median percentage values. Mann–Whitney–Wilcoxon test, ns, not significant, **P< 0.01.

In a previous study, we documented the tolerance of Polϵ-exonuclease-deficient chicken DT40 and human TK6 B lymphoid cells (*POLE1^exo^*^−/−^) cells to cisplatin, UV, ICRF193 (a DNA topoisomerase II inhibitor), γ-rays, and olaparib (38). Hypersensitivity to CPT was previously demonstrated in a yeast mutant carrying dysregulated Polϵ exonuclease activity (46). Therefore, here, we analyzed the sensitivity of our human *POLE1^exo^*^−/−^ cell lines to CPT. The results showed that despite the tolerance of *POLE1^exo^*^−/−^ DT40 and TK6 cells to olaparib, these cells showed significantly increased sensitivity to CPT (Figure 1A, B). Both of these agents kill cancer cells by generating excessive SSBs (reviewed in (2,7), so the differential sensitivity of *POLE1^exo^*^−/−^ cells to CPT and olaparib was unexpected. As olaparib inhibits PARP1-dependent FR, we hypothesized that Polϵ exonuclease activity may function in PARP1-dependent FR at broken template strands.

As expected from the CPT hypersensitivity of *POLE1^exo^*^−/−^ DT40 and TK6 cells, mitotic chromosome spreads prepared from these cells exhibited increased numbers of chromosome aberrations (CAs), compared with wild-type cells, following CPT exposure (Figure 1C, D, Supplementary Figure S3A). We confirmed the enhanced production of CPT-induced DSBs in *POLE1^exo^*^−/−^ cells using PFGE (Figure 1E) and neutral comet assays (Figure 1F, G). These results suggest that Polϵ exonuclease may suppress CPT-induced DSBs by promoting HDR or FR (Supplementary Figure S1).

### Proficient HDR in CPT-treated *POLE1^exo^*^−/−^ cells

**Figure 2.**
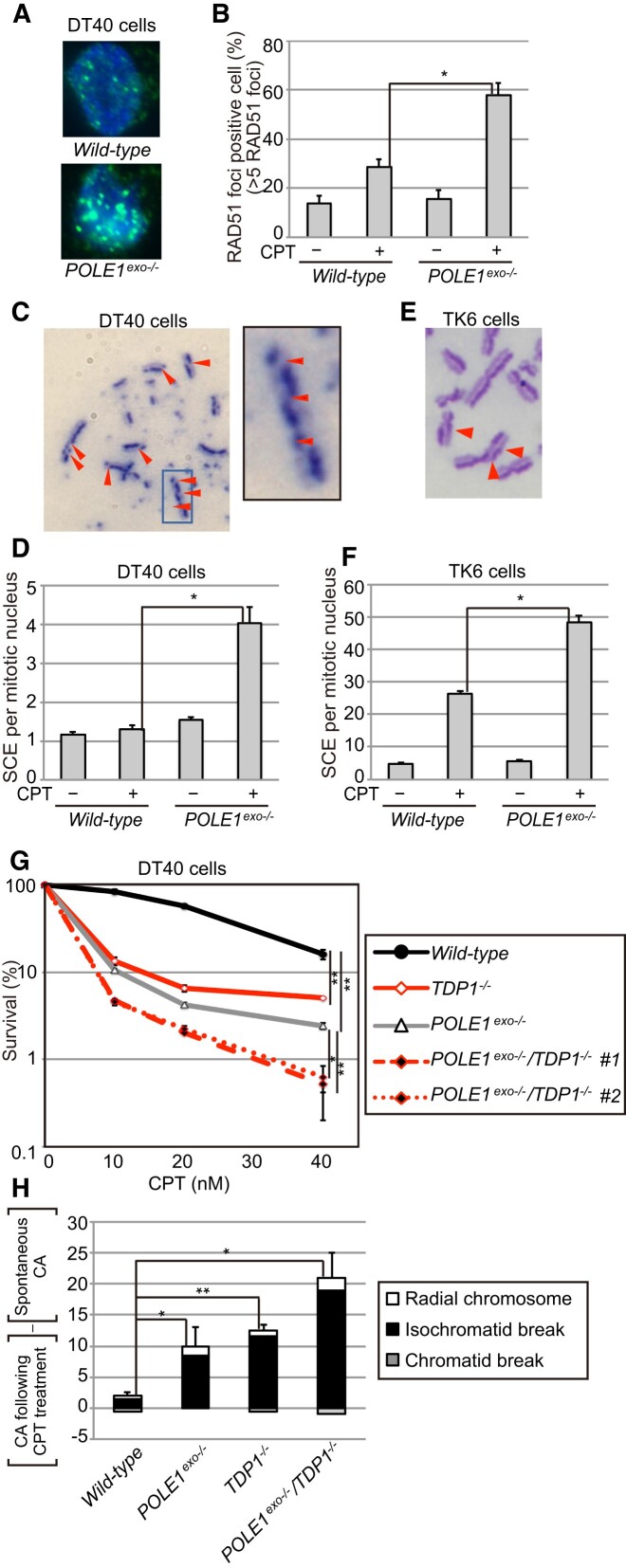
Polϵ proofreading exonuclease activity acts independently of HDR and TDP1-mediated Top1ccs repair. (**A**) Representative images showing RAD51 foci in cells treated with CPT (50 nM) for 4 h. (**B**) Quantification of the RAD51 foci-positive cell fraction. Cells with >5 RAD51 foci per cell were defined as RAD51 foci-positive. Numbers of RAD51 foci per 100 nuclei were scored for each genotype. The error bars indicate the SD of two independent analyses. (C–F) Analysis of sister chromatid exchanges (SCEs) in DT40 (**C, D**) and TK6 (E, F) cells treated with CPT (2 nM) and BrdU for 16 h or CPT (0.5 nM) and BrdU for 24 h, respectively. Representative images are shown in (C) and (**E**); red arrowheads indicate the positions of SCEs. Numbers of SCEs were scored for 50 mitotic nuclei per experiment. Data in (D) and (**F**) represent means ± standard deviation (SD) from two independent experiments. (**G**) CPT sensitivities of DT40 cells with the indicated genotypes. Data represent means ± SD from two independent experiments; Student's *t*-test, *P< 0.05 and ** *P*< 0.01. (**H**) CPT-induced chromosomal aberrations (CAs); the number of CAs in untreated cells was subtracted from the number in CPT-treated cells. DT40 cells with the indicated genotypes were treated with CPT (20 nM) for 8 h with the addition of colcemid for the last 3 h. The numbers of CAs per 50 mitotic nuclei before and after CPT treatment were scored in two independent analyses (data for the individual experiments are shown in Supplementary Figure S3B). Average CA numbers and SDs from two independent studies are shown in the bar graph. Student's *t*-test, *P< 0.05 and ** *P*< 0.01.

In order to exclude the possibility that the loss of Polϵ exonuclease may have resulted in the suppression of HDR, we examined HDR functionality in *POLE1^exo^*^−/−^ cells by measuring CPT-induced RAD51 foci (67,68) and sister chromatid exchanges (SCEs) (69). The results showed higher levels of CPT-induced RAD51 foci in *POLE1^exo^*^−/−^ cells than in wild-type cells (Figure 2A, B). In addition, the *POLE1^exo^*^−/−^ mutation increased the number of CPT-induced SCEs in DT40 cells (Figure 2C, D) and TK6 cells (Figure 2E, F). These data indicate proficient HDR in *POLE1^exo^*^−/−^ cells and support the role of Polϵ exonuclease activity in FR (Supplementary Figure S1, step 2) rather than HDR (Supplementary Figure S1, steps 3 to 5).

### Polϵ exonuclease activity contributes to CPT tolerance independently of TDP1-mediated TOP1ccs repair

TDP1 promotes the repair of CPT-induced SSBs by removing 3′ TOP1 adducts, preventing seDSB formation (27,28). Therefore, we examined the functional relationship between Polϵ exonuclease and TDP1 by creating *POLE1^exo^*^−/−^/*TDP1*^−/−^ DT40 cells. Combined *POLE1^exo^*^−/−^ and *TDP1*^−/−^ mutations displayed an additive effect on both cell survival (Figure 2G) and CPT-induced CAs (Figure 2H, Supplementary Figure S3B). This additive effect indicates that Polϵ proofreading exonuclease activity and TDP1-mediated repair contribute independently to CPT tolerance.

### Polϵ exonuclease is necessary for fork slowing upon CPT treatment

**Figure 3.**
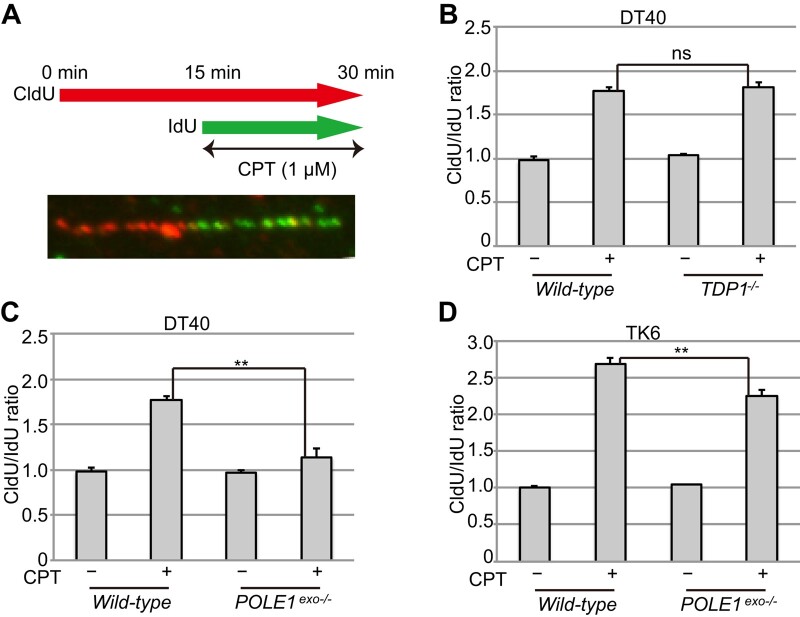
The proofreading exonuclease activity of Polϵ is required for fork slowing in CPT-treated cells. (A–D) The speed of replication fork progression before and after CPT treatment. A schematic of the DNA fiber analysis method and a representative image are shown in (**A**). DT40 cells with the indicated genotypes were labeled sequentially with CldU and IdU for 15 min each and treated with CPT (1 μM) during the IdU labeling step. The lengths of the CldU and IdU tracts were measured, and the CldU/IdU ratio in each replication fork was calculated for at least 100 replication forks (**B, C**). Data represent means ± SD from medians in two independent experiments. Student's *t*-test, ns, not significant, and *P< 0.05. (Histograms with the distribution of CldU/IdU ratios for individual replication forks in the two experiments are shown in Supplementary Figure S4). In (**D**), TK6 cells with the indicated genotypes were labeled sequentially with CldU and IdU for 20 min each and treated with CPT (1 μM) during the IdU labeling step. Data represent means ± SE from at least 120 replication forks. Student's *t*-test, **P< 0.01.

Having shown that Polϵ exonuclease plays a crucial role in suppressing CPT-induced seDSBs independent of HDR and TDP1-mediated repair, we went on to explore the role of Polϵ exonuclease in FR at broken template DNA. Previous studies established that FR can be detected by using DNA fiber assays to identify fork slowing upon the addition of CPT (Figure 3A) (15–20). In these assays, fork slowing is indicated by an increased CldU/IdU ratio caused by a decrease in the length of nascent DNA tracts during the CPT treatment/IdU pulse-labeling step (15–20) (Figure 3A). Without CPT treatment, the CldU/IdU ratio was approximately 1.0 in both wild-type and *POLE1^exo^*^−/−^ cells. As expected, after CPT treatment, the speed of replication was significantly reduced (*P*< 0.05), with the average CldU/IdU ratio increasing to approximately 1.9 in wild-type cells (Figure 3B, C, Supplementary Figure S4A). The loss of TDP1 did not further delay fork progression (Figure 3B, Supplementary Figure S4B), indicating that the extent of fork slowing does not reflect the number of stalled TOP1ccs.

In sharp contrast to *TDP1*^−/−^ cells, *POLE1^exo^*^−/−^ cells exhibited longer DNA replication tracts after CPT treatment than wild-type cells, with the average CldU/IdU ratio only increasing to approximately 1.3 (Figure 3C, Supplementary Figure S4A, S4B). Polϵ exonuclease inactivation also impaired fork slowing in human TK6 cells, with CPT treatment increasing the average CldU/IdU ratio to approximately 2.7 in wild-type cells and 2.2 in *POLE1^exo^*^−/−^ cells (Figure 3D). These data indicate that fork slowing in DT40 and TK6 cells requires Polϵ exonuclease activity. CPT treatment is also known to increase the percentage of asymmetric forks due to R-loop formation (15–20); however, our analysis showed that the percentages of asymmetric forks were increased to the same extent in wild-type and *POLE1^exo^*^−/−^ cells following CPT treatment (Supplementary Figure S5). We conclude that Polϵ exonuclease likely facilitates CPT tolerance by mediating FR (Supplementary Figure S1, Step 2).

### Polϵ exonuclease requires PARP1 for cellular tolerance to CPT

**Figure 4.**
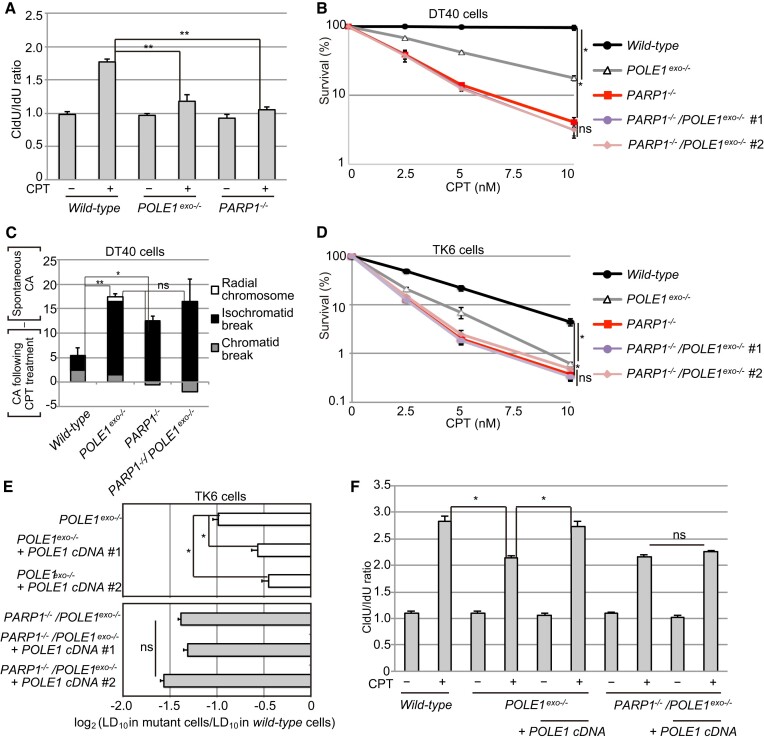
Epistatic relationship between *POLE1^exo^*^−/−^ and *PARP1^−^*^/−^ in cellular tolerance to CPT. (**A**) DNA fiber analysis was performed in DT40 cells with indicated genotype as in Figure 3A. Data represent means ± SD from medians in two independent experiments; Student's *t*-test, ns, not significant, and *P< 0.05. (Histograms with the distribution of CldU/IdU ratios for individual replication forks in the two experiments are shown in supplementary Figure S4). Student's *t*-test, **P< 0.01. (CldU/IdU ratios of each replication fork are shown in Supplementary Figure S4A; the degree of fork asymmetry is shown in Supplementary Figure S5.) (**B**) The CPT sensitivity of DT40 cells with the indicated genotypes was analyzed. Data represent means ± SD from two independent experiments; Student's *t*-test, *P< 0.05 and ns, not significant. (**C**) CPT-induced chromosomal aberrations (CAs) were analyzed and shown as in Figure 2H. (The results of the two independent analyses are shown in supplementary Figure S3C.) Student's *t*-test, *P< 0.05 and **P< 0.01. (D, E) The CPT sensitivity of the indicated TK6 cells is shown as in Figure 1B (**D**). The bar graph in (**E**) indicates the relative sensitivity of each type of mutant TK6 cell line, compared with wild-type cells, which was scored as log2 (LD_10_ in the indicated mutant cells/LD_10_ in wild-type cells); LD_10_ represents the concentration of CPT that reduces survival to 10%. Student's *t*-test *P< 0.05. (**F**) DNA fiber analysis was performed as in Figure 3D. Data represent means ± SD from medians in two independent experiments; Student's *t*-test, *P< 0.05.

The role of Polϵ exonuclease in CPT-induced fork slowing is reminiscent of the role played by PARP1 (18,27,28,70). We, therefore, explored the functional relationship between Polϵ-exonuclease and PARP1 by generating *POLE1^exo^*^−/−^/*PARP1^−^*^/−^ cells. Consistent with previous data (18,27,28,70), the loss of PARP1 alone in DT40 cells impaired fork slowing upon CPT treatment (Figure 4A, Supplementary Figure S4A) and caused CPT hypersensitivity (Figure 4B). Strikingly, *PARP1^−^*^/−^ and *POLE1^exo^*^−/−^/*PARP1^−^*^/−^ cells displayed indistinguishable levels of CPT hypersensitivity (Figure 4B). Consistent with these findings, CPT induced similar numbers of CAs in *PARP1^−^*^/−^ and *POLE1^exo^*^−/−^/*PARP1^−^*^/−^ cells (Figure 4C, Supplementary Figure S3C). This epistatic relationship between *PARP1^−^*^/−^ and *POLE1^exo^*^−/−^ was also observed in human TK6 cells (Figure 4D).

Next, we tested the effect of expressing intact *POLE1* cDNA in both *POLE1^exo^*^−/−^ cells and *PARP1^−^*^/−^/*POLE1^exo^*^−/−^ cells. Consistent with the epistasis data, ectopic *POLE1* expression reversed hypersensitivity to CPT in *POLE1^exo^*^−/−^ cells but not in *PARP1^−^*^/−^/*POLE1^exo^*^−/−^ cells (Figure 4E). The rescue of CPT-mediated fork slowing by ectopic *POLE1* expression was also restricted to cells expressing PARP1 (Figure 4F). Taken together, the observed epistatic relationship and rescue experiment results indicate that PARP1 is essential for the functionality of Polϵ exonuclease in both CPT tolerance and fork slowing upon CPT treatment. Therefore, we propose that the exonuclease activity of Polϵ plays a role in PARP1-mediated FR at CPT-induced broken templates.

### PARP1 is not required for the cellular response to aphidicolin and hydroxyurea

Previous studies examined mechanisms for FR upon replication blockage by exposing cells to aphidicolin (an inhibitor of replicative DNA polymerases) and hydroxyurea. To determine whether PARP1 contributes to the response to aphidicolin and hydroxyurea, we analyzed the sensitivities of *POLE1^exo−/−^*, *PARP1^−/−^*, and *POLE1^exo−/−^/PARP1^−/−^* cells to these agents. The results showed that *PARP1^−/−^* cells showed no significant sensitivity to aphidicolin or hydroxyurea (Supplementary Figure S2F). These findings indicated that PARP1 does not contribute to tolerance to either of these chemicals. By contrast, *POLE1^exo−/−^* cells showed higher sensitivity to aphidicolin and hydroxyurea than wild-type cells. This hypersensitivity is unlikely to result from the same mechanism as the CPT hypersensitivity of *POLE1^exo−/−^* cells because of the differing mechanisms by which CPT and aphidicolin/hydroxyurea interfere with DNA replication. Thus, we conclude that the collaboration between PARP1 and Polϵ exonuclease does not facilitate the response to the inhibition of DNA synthesis via replicative DNA polymerases.

### PARP1 is required for the functionality of Polϵ exonuclease and TDP1 in cellular tolerance to CPT

The above data indicated that the CPT sensitivity of *PARP1*^−/−^ cells was higher than that of *POLE1^exo^*^−/−^ cells (Figure 4B, Supplementary Figure S6A). Thus, PARP1 also promotes tolerance to CPT independent of Polϵ exonuclease activity. To investigate this independent role, we analyzed the functional relationship between PARP1 and TDP1, as PARP1 is required for the TDP1-dependent removal of 3′ TOP1 adducts from SSBs (29). The results confirmed that *PARP1*^−/−^ and *PARP1*^−/−^*/TDP1*^−/−^ cells showed very similar CPT sensitivity, verifying this relationship (Supplementary Figure S6A).

Next, we investigated whether the regulation of pathways other than Polϵ exonuclease activity and TDP1 by PARP1 might contribute to CPT tolerance. *PARP1*^−/−^ and *POLE1^exo^*^−/−^/*TDP1*^−/−^ cells showed similar levels of CPT sensitivity (Supplementary Figure S6A). Moreover, *PARP1*^−/−^, *PARP1*^−/−^*/TDP1*^−/−^, *PARP1^−^*^/−^*/POLE1^exo^*^−/−^, and *PARP1*^−/−^*/POLE1^exo^*^−/−^/*TDP1*^−/−^ cells showed similar sensitivities to CPT (Supplementary Figure S6B). These genetic data indicated that PARP1-mediated regulation of Polϵ exonuclease and TDP1, but not other pathways, underlies cellular tolerance to CPT. These dual roles of PARP1 explain the extremely high CPT sensitivity of PARP1-deficient cells (Supplementary Figure S2A).

### Collaboration of Polϵ exonuclease and CTF18 in CPT tolerance

We next searched for factors that might collaborate with Polϵ exonuclease in mediating cellular tolerance to CPT. For this purpose, we analyzed the sensitivity profile of a comprehensively gene-disrupted RPE1 cell library in response to 28 DNA-damaging agents (12) (Supplementary Figure S2B to E). We identified the clamp loader CTF18, a component of the leading-strand replisome (47,48), as a promising candidate. The loss of CTF18 sensitized RPE1 cells to CPT but not to olaparib, cisplatin, or hydroxyurea (Supplementary Figure S2B–E), similar to the effects of inactivating PARP1 activity (38) (Figure 4B, Supplementary Figure S2B–E). These data suggest that CTF18 plays a role in PARP1-dependent fork protection of broken template strands but not in HDR.

**Figure 5.**
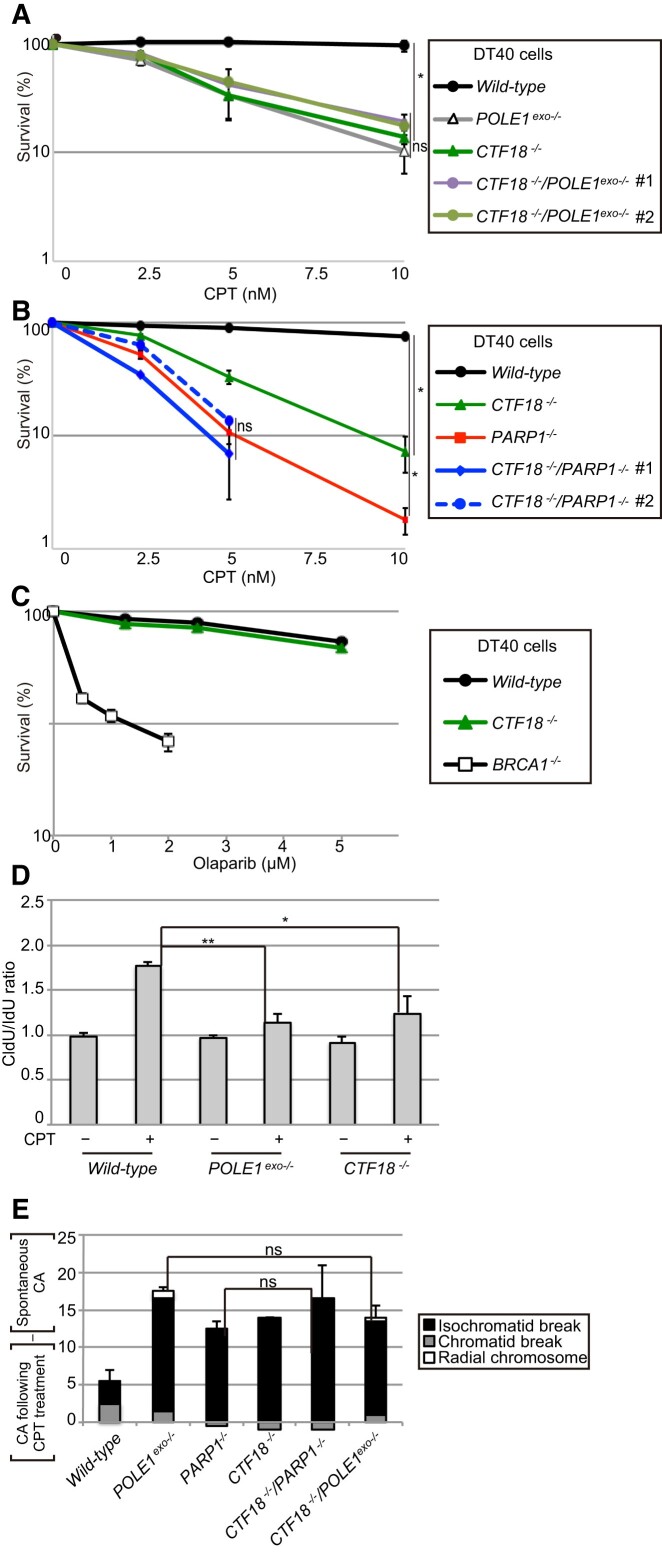
The *PARP1*^−/−^ genotype is epistatic to both *POLE1^exo^*^−/−^ and *CTF18^−^*^/−^ in cellular tolerance to CPT. (A–C) Analysis of the sensitivity of DT40 cells with the indicated genotypes to CPT (**A, B**) and olaparib (**C**). Student's *t*-test, *P< 0.05 and ns, not significant. Analysis and data presentation are as in Figure 1A. (**D**) DNA fiber analysis was performed as in Figure 3A. Data represent means ± SD from medians in two independent experiments; Student's *t*-test, **P< 0.01. (The CldU/IdU ratios of individual replication forks are shown in Supplementary Figure S4; the degree of fork asymmetry is shown in Supplementary Figure S5.) (**E**) CPT-induced chromosomal aberrations (CAs) were analyzed and shown as in Figure 2H. (The results of the two independent analyses are shown in Supplementary Figure S3C.)

We then conducted a phenotypic analysis of *PARP1^−/−^*, *CTF18*^−/−^/*POLE1^exo^*^−/−^ and *CTF18*^−/−^/*PARP1^−/−^* cells. The results showed that the loss of CTF18 in DT40 cells caused hypersensitivity to CPT (Figure 5A, B) but not to olaparib (Figure 5C). Similar to *POLE1^exo^*^−/−^ and *PARP1^−^*^/−^ cells (Figures 1A, B and 4B), *CTF18*^−/−^ cells showed impaired fork slowing, with a median CldU/IdU ratio of approximately 1.3 (Figure 5D, Supplementary Figure S4A). In addition, *CTF18*^−/−^, *POLE1^exo^*^−/−^ and *CTF18*^−/−^/*POLE1^exo^*^−/−^ cells showed similar CPT sensitivities and indistinguishable levels of CPT-induced CAs (Figure 5A, E, Supplementary Figure S3C). These data indicated that CTF18 and Polϵ-exonuclease have interdependent roles in cellular tolerance to CPT. Similarly, *PARP1^−/−^* and *CTF18*^−/−^/*PARP1^−/−^* cells displayed similar phenotypes in terms of CPT sensitivity (Figure 5B) and CPT-induced CAs (Figure 5E, Supplementary Figure S3C). Overall, the *PARP1^−/−^* genotype was found to be epistatic to both *POLE1^exo^*^−/−^ and *CTF18*^−/−^. These data support the involvement of the PARP1-CTF18-Polϵ exonuclease axis in preventing fork collapse at broken templates.

### Polϵ-exonuclease and PARP1 antagonize RECQ1 to mediate fork slowing upon CPT treatment

**Figure 6.**
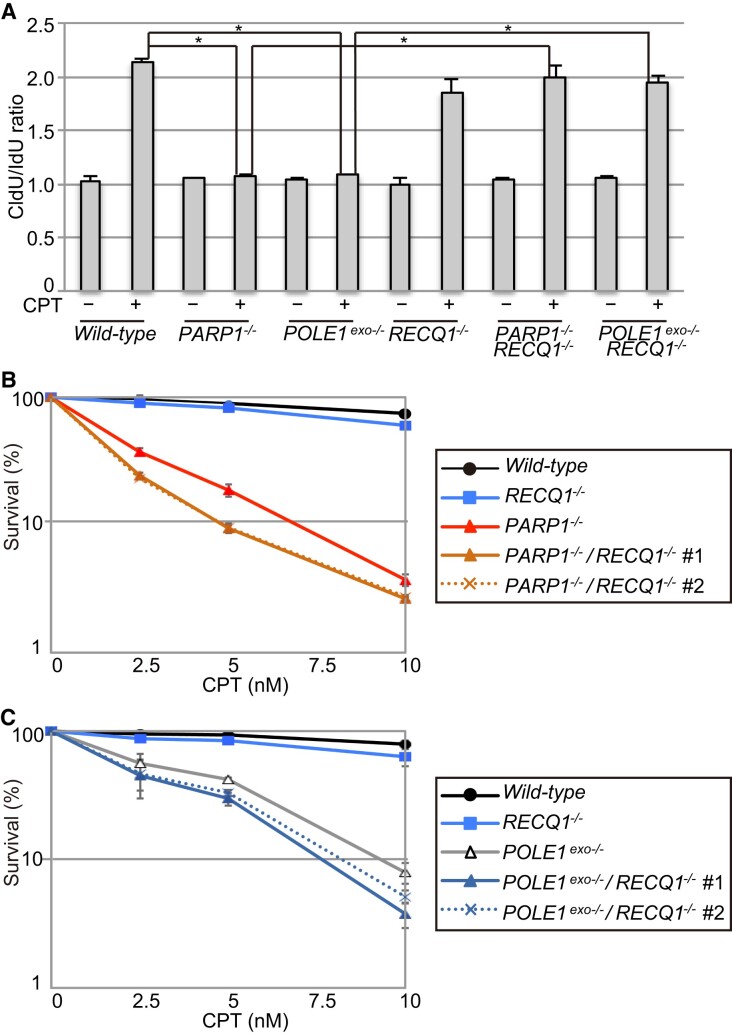
Polϵ-exonuclease and PARP1 antagonize RECQ1-mediated FR resolution. (**A**) DNA fiber analysis in DT40 cells with the indicated genotypes was performed as in Figure 3A. Data represent means ± SD from medians in two independent experiments; Student's *t*-test **P< 0.01. (The CldU/IdU ratios of individual replication forks are shown in Supplementary Figure S7; the degree of fork asymmetry is shown in Supplementary Figure S4.) (**B, C**) CPT sensitivity of DT40 cells with the indicated genotypes. Analysis and data presentation are as in Figure 1A.

Given the role of RECQ1 in counteracting PARP1-dependent FR, we next investigated the functional interaction of Polϵ exonuclease with RECQ1 (15,18,20–22). In accordance with its repressive role, the loss of RECQ1 completely restored the impaired fork slowing observed in *POLE1^exo^*^−/−^ and *PARP1^−^*^/−^ cells (Figure 6A, Supplementary Figure S7). Nonetheless, the loss of RECQ1 did not rescue the CPT hypersensitivity of *POLE1^exo^*^−/−^ and *PARP1^−^*^/−^ cells (Figure 6B, C). These data support the notion that both PARP1 and Polϵ exonuclease induce fork slowing by counteracting the action of RECQ1 in the resolution of FR. However, PARP1 and Polϵ exonuclease are still required for cellular survival upon CPT treatment, even without RECQ1.

### The exonuclease activity of Polϵ compensates for the lack of BRCA1

**Figure 7.**
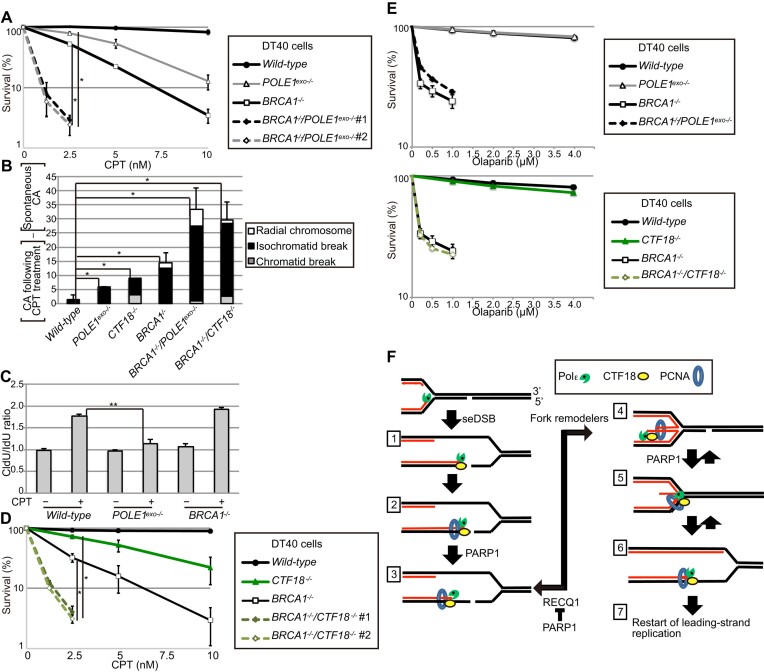
*BRCA1* and *POLE1^exo^*^−/−^ mutations have a synergistic impact on CPT sensitivity. (A, D, E) Sensitivity of DT40 cells with the indicated genotypes to CPT (**A, D**) or olaparib (**E**). Analysis and data presentation are as in Figure 1A. Student's *t*-test, *P< 0.05. (**B**) CPT-induced chromosome breaks. DT40 cells with the indicated genotypes were treated with CPT (10 nM) for 8 h with the addition of colcemid for the last 3 h. The data are presented as in Figure 2H. (The results of the two independent analyses are shown in Supplementary Figure S3D.) (**C**) DNA fiber analysis was performed as in Figure 3A. Data represent means ± SD from medians in two independent experiments; Student's *t*-test, **P< 0.01, ns, not significant. (The CldU/IdU ratios of individual replication forks are shown in Supplementary Figure S4.) (**F**) A model for the role of the PARP1-CTF18-Polϵ exonuclease axis in inhibiting Polϵ run-off at the edge of seDSBs. CTF18 mediates the tethering of Polϵ at the stalled end (steps 1 to 2). The tethering of Polϵ facilitates Polϵ exonuclease-mediated resection of the 3′ end of the nascent leading strand (steps 2 to 5), keeping Polϵ away from the seDSBs until the stalled TOP1ccs are repaired (step 5). PARP1 might be involved in the activation of Polϵ exonuclease-mediated resection.

Our results showing that *POLE1^exo^*^−/−^ cells displayed increased HDR of DSBs following exposure to CPT (Figure 2A–F) suggested that Polϵ exonuclease and HDR may complement each other in CPT tolerance (Supplementary Figure S1). This concept led us to investigate the impact of HDR deficiency in *POLE1^exo^*^−/−^ cells. As DT40 cells proliferate in the complete absence of BRCA1, a pivotal factor for HDR (71,72), we disrupted the *BRCA1* gene in *POLE1^exo^*^−/−^ DT40 cells. Remarkably, the CPT sensitivities of *BRCA1*^−/−^ and *POLE1^exo^*^−/−^ cells were comparable while combining these mutations synergistically increased CPT sensitivity (Figure 7A). Similarly, combining *BRCA1*^−/−^ and *POLE1^exo^*^−/−^ mutations synergistically increased the numbers of CPT-induced chromosomal breaks (Figure 7B, Supplementary Figure S3D). DNA fiber assays showed that *BRCA1*^−/−^ cells were proficient in fork slowing (Figure 7C), supporting the view that HDR may not be involved in FR (Supplementary Figure S1). Moreover, similar to *BRCA1*^−/−^/*POLE1^exo^*^−/−^ mutants, *BRCA1*^−/−^/*CTF18*^−/−^ cells displayed much more severe phenotypes than *BRCA1*^−/−^ or *CTF18*^−/−^ cells (Figure 7B and D, Supplementary Figure S3D). Taken together, these results indicate that Polϵ exonuclease can compensate for defective HDR of DSBs caused by CPT. Thus, our data revealed synthetic lethality between HDR and *POLE1^exo^*^−/−^ mutations in the presence of excessive SSBs.

We then assessed the olaparib sensitivity of wild-type, *BRCA1*^−/−^, *BRCA1*^−/−^/*POLE1^exo^*^−/−^ cells and *BRCA1*^−/−^/*CTF18*^−/−^ cells. Although *BRCA1*^−/−^/*POLE1^exo^*^−/−^ and *BRCA1*^−/−^/*CTF18*^−/−^ cells showed synthetic lethality in response to CPT (Figure 7A and D), *BRCA1*^−/−^, *BRCA1*^−/−^/*POLE1^exo^*^−/−^ cells, and *BRCA1*^−/−^/*CTF18*^−/−^ cells showed comparable sensitivity to olaparib at clinical concentrations (0.1 to 1 μM) (73) (Figure 7E). We concluded that olaparib effectively suppresses the PARP1-CTF18-Polϵ exonuclease axis.

## Discussion

This genetic study identified the PARP1-CTF18-Polϵ exonuclease axis as a critical pathway for preventing fork collapse at broken template strands. Moreover, our data suggest that PARP1 controls the activity of CTF18, Polϵ exonuclease, and TDP1 (Figures 4, 5 and Supplementary Figure S6). This relationship underlies the hypersensitivity of PARP1-deficient cells to CPT (12) (Supplementary Figure S2A), while the dependence of CTF18 and Polϵ exonuclease on PARP1 explains why cells deficient in these factors are tolerant to olaparib (Figures 1A, B, 5C, 7E). We also demonstrated the synthetic lethality of defects in HDR and the CTF18-Polϵ exonuclease axis in cells treated with CPT (Figure 7). We propose that olaparib is synthetically lethal with defective HDR because olaparib not only generates excessive SSBs (51,52,74, and reviewed in 2,7) but also suppresses the PARP1-CTF18-Polϵ exonuclease axis.

Based on our epistasis analysis, we propose a model for the role of the PARP1-CTF18-Polϵ exonuclease axis in preventing fork collapse (Figure 7F). The unexpected role of Polϵ exonuclease in preventing seDSBs allows the DNA damage response during leading-strand replication to be distinguished from that during lagging-strand replication. The function of CTF18 may be to tether Polϵ tightly at the replisome (48,75) via PCNA loading at the end of the broken template (76) (Figure 7F, step 2). Importantly, PARylation of purified Polϵ by PARP1 reduces its DNA synthesis rate by approximately 10-fold (77). The reduced synthesis rate and robust 3′–5′ exonucleolytic activity of Polϵ (38,45) may keep Polϵ away from seDSBs, preventing Polϵ run-off. When exonuclease activity exceeds DNA synthesis, Polϵ digests nascent DNA (Figure 7F, step 3), and the resulting single-stranded DNA facilitates template strand reannealing, leading to FR (Figure 7F, step 4). The occurrence of FR in cells proficient in the PARP1-CTF18-Polϵ exonuclease axis (Figure 6A, B) is supported by our data showing that *RECQ1^−/−^* mutation in *POLE1^exo^*^−/−^ and *PARP1^−^*^/-^ cells restored their impaired fork slowing (Figure 6A, B). This restoration is consistent with the established role of RECQ1 in counteracting PARP1-dependent FR (Figure 7F, steps 3 to 4) (15,18,20–22). Nonetheless, the loss of RECQ1 did not rescue the CPT hypersensitivity of *POLE1^exo^*^−/−^ and *PARP1^−^*^/-^ cells (Figure 6B, C), suggesting that the PARP1-CTF18-Polϵ exonuclease axis may also play a crucial role in steps other than FR. In steps 1 to 6 (Figure 7F), we propose that the PARP1-CTF18-Polϵ exonuclease axis prevents Polϵ run-off via the inhibition of Polϵ-mediated DNA synthesis. Loss of Polϵ exonuclease activity increases the number of collapsed replication forks and unrepaired DSBs, which reduces cellular viability as a single unrepaired DSB can trigger apoptosis. In summary, the newly identified PARP1-CTF18-Polϵ exonuclease axis ensures the tethering of the stalled Polϵ to template strands on the edge of seDSBs.

In our functional and mechanistic studies, we utilized the chicken DT40 cellular model. The phenotypes of the human TK6 and chicken DT40 Polϵ proofreading mutants were similar following CPT treatment (Figures 1, 3), indicating that the roles of the proofreading exonuclease in preventing seDSBs have been evolutionarily conserved. DT40 cells showed a more prominent phenotype than TK6 cells (Figure 1), potentially due to their lack of functional p53 (78,79), as well as increased replication stress caused by the overexpression of c-Myc (80,81).

Yeast genetic studies indicate that proofreading defects increase the number of misincorporated ribonucleotides (39–41), which efficiently trap TOP1, generating TOP1ccs (82–84). It could, thus, be speculated that the CPT hypersensitivity in *POLE1^exo^*^−/−^ cells is caused by augmented TOP1cc formation. However, compared with yeast species, the impact of misincorporated ribonucleotides on seDSB formation in metazoan cells during the subsequent round of replication should be much smaller. Metazoan cells have a longer cell cycle time, while human TDP1 removes 90% of trapped TOP1 within 15 min (85). Therefore, misincorporated ribonucleotides are unlikely to generate seDSBs.

Our data revealed that *POLE1^exo^*^−/−^ and *CTF18*^−/−^ cells allow HDR to be distinguished from PARP1-dependent fork protection, including FR, at broken template strands. Importantly, the synergistic effects of *BRCA1*^−/−^ and *POLE1^exo^*^−/−^ or *CTF18*^−/−^ on CPT sensitivity (Figure 7) suggest that HDR does not play a significant role in FR at broken template strands. This synthetic lethality has not been demonstrated previously as PARP1 has multiple roles in the DNA damage response besides its role in FR, and previous studies have failed to identify the factors that are specifically involved in PARP1-dependent fork protection (24,86,87). Previous studies indicated that the stability of reversed forks depended on HDR factors, including RAD51 and its cofactors, such as BRCA2 (14,88). Most of these studies examined FR generated by the complete blockade of replication fork progression caused by hydroxyurea treatment for >1 h (14,88); however, it is unlikely that HDR factors are able to assemble at stalled forks within 15 min of the start of CPT treatment and thereby slow fork progression (Figure 3A). Given the synthetic lethality caused by concurrent defects in HDR and the PARP1-CTF18-Polϵ exonuclease axis, we propose that the latter pathway works independently of HDR.

PARP inhibitors are highly effective against HDR-deficient cancers, but resistance eventually develops in most cases (89). Although both CPT and PARP inhibitors are thought to kill cancer cells by generating seDSBs (90), we verified that *POLE1^exo^*^−/−^ and *CTF18*^−/−^ cells were tolerant to olaparib (Figure 7E) because of its suppression of the PARP1-Polϵ exonuclease axis. This concept is supported by the finding that veliparib (ABT-888), a PARP inhibitor that only modestly inhibits SSB repair (90), significantly increases the cytotoxic effect of CPT (25). We propose that chemical compounds targeting the exonuclease activity of Polϵ may synergistically enhance the effects of CPT treatment and might also reverse acquired resistance to olaparib. The molecular mechanisms of CPT-induced FR are poorly understood compared with those induced by hydroxyurea. Exploring the intricate mechanisms of replication fork protection at SSBs may provide important clues to improving cancer chemotherapy using TOP1 poisons and clinical PARPi.

### Limitations of the current study

While our genetic approach uncovered the functional relationship of PARP1 with CTF18 and Polϵ exonuclease, we have not yet clarified the mechanism of PARP1-dependent activation of the CTF18-Polϵ exonuclease axis. This study did not demonstrate the functionality of the exonuclease activity or the resulting tethering of stalled Polϵ to template strands (Figure 7F, steps 1 to 3 and 4 to 6). In addition, while the loss of proofreading activity significantly reduced cell viability upon CPT treatment, neither the number of Polϵ molecules in the chromatin fraction nor the number of replication protein A foci (data not shown) was reduced during exposure to CPT. However, these findings are unsurprising as the numbers of Polϵ run-off events and resulting DSBs are likely to be limited even in *POLE1^exo^*^−/−^ cells because a single unrepaired DSB can trigger apoptosis. The limited number of Polϵ run-off events is supported by our data showing that the *POLE1^exo^*^−/−^ mutation increased the number of chromosomal breaks by <10 per 100 mitotic cells following CPT treatment (Figure 1B, C). Thus, the vast majority of Polϵ molecules would be expected to remain at stalled replication forks at seDSBs in *POLE1^exo^*^−/−^ cells, explaining why we failed to detect diminished tethering of stalled Polϵ to template strands in these cells. We propose that the proofreading activity forces Polϵ away from the edge of seDSBs and does not perform extensive degradation of nascent strands, which may explain why we failed to detect augmentation of single-strand gap formation at replication foci. Further studies are required to identify the substrates of PARylation in this process and the effects of PARylation on the exonuclease activity and retention of Polϵ *in vivo*.

## Supplementary Material

gkad999_Supplemental_File

## Data Availability

The data underlying this article are available in the article and in its online supplementary material.
