# Supplementary material for: The proofreading exonuclease of leading-strand DNA polymerase epsilon prevents replication fork collapse at broken template strands
Source: Nucleic Acids Res. 2023 Nov 8;51(22):12288–302. doi: 10.1093/nar/gkad999 (PMC10711444; doi:10.1093/nar/gkad999)
Supplement: gkad999_Supplemental_File [file gkad999_supplemental_file.pdf]

Supplementary Information

The proofreading exonuclease of leading-strand DNA polymerase epsilon prevents replication fork collapse at broken template strands

Tasnim Ahmad, Ryotaro Kawasumi, Tomoya Taniguchi, Takuya Abe, Kazuhiro Terada, Masataka Tsuda, Naoto Shimizu, Toshiki Tsurimoto, Shunichi Takeda, and Kouji Hirota

Supplementary figure legends

Fig. S1. Mechanism of fork protection induced by replication fork stalling at broken templates

Schematic showing the sequence of events that occurs when a leading-strand DNA replication fork encounters an SSB. First, an seDSB arises (step 1); then, FR (step 2) and HDR (steps 3 to 5) prevent fork collapse and restart DNA replication (step 6). The yellow circle represents TOP1 covalently attached to the 3' end of the SSB. We propose that the PARP1-CTF18-Polε exonuclease axis facilitates steps 2 and 6, while previous studies have established an inhibitory function for RECQ1 in step 2.

Fig. S2 *PARP1*$^{-/-}$ and *CTF18*$^{-/-}$ cells show high sensitivity to CPT but not to cisplatin

(A) Sensitivity profiles of PARP1/PARP2-deficient RPE cells to the DNA-damaging drugs shown to the left of the two panels. The x-axis represents the relative sensitivity of these mutant cells relative to that of wild-type cells calculated from an open database (77). Relative sensitivity is scored as $\log_2$ (LD20% in the indicated mutant cells)/(LD20% in wild-type cells); LD20% represents the drug concentration that reduces cell survival to 20% relative to untreated cells. Note that sensitivity was measured twice for CPT and three times for cisplatin (77). Negative (left) and positive (right) scores indicate that the indicated gene disruption induces sensitivity and tolerance, respectively, to the indicated DNA-damaging agents. (B to E) Blue dot plots

show the relative sensitivity to CPT (x-axis) and the indicated drugs (y-axis) of RPE clones deficient in individual genes. The y-axes show the relative sensitivity to acute exposure to hydroxyurea (HU) (B), chronic exposure to HU (C), and exposure to olaparib (D) or cisplatin (E). Red dots show cells deficient in the indicated genes. (F) Sensitivity of DT40 cells with the indicated genotypes to hydroxyurea or aphidicolin. Analysis and data presentation are as in Figure 1A.

Fig. S3. Quantification of chromosome breaks induced by CPT treatment

(A–D) DT40 cells with the indicated genotypes were treated with CPT as indicated. Data represent the mean ± SE from at least 50 mitotic nuclei.

Fig. S4. Distribution of CldU/IdU ratios for individual replication forks following CPT treatment

(A, B) DT40 cells with the indicated genotypes were labeled sequentially with CldU and IdU for 15 min each and treated with CPT (1 μM) at the same time as the IdU labeling. The lengths of the CldU and IdU tracts were measured, and the CldU/IdU ratios were calculated for at least 100 replication forks. Wild-type, *POLE1*$^{exo-/-}$, *PARP1*$^{-/-}$, and *CTF18*$^{-/-}$ clones were examined twice independently (A). Wild-type, *POLE1*$^{exo-/-}$, *TDP1*$^{-/-}$, and *BRCA1*$^{-/-}$ clones were examined twice independently (B). Medians and SEs for each test are indicated in the boxes.

Fig. S5. Analysis of fork asymmetry following CPT treatment

(A) Representative images showing bi-directionally elongating forks. DT40 cells with the indicated genotypes were labeled sequentially with CldU and IdU for 15 min each and treated with CPT (1 μM) during the IdU labeling step. (B) Quantification of the green segments (labeled second). The lengths of the (green) replication tracts generated on the left and right of the images during the 15 min of labeling were plotted for the indicated genotypes. At least 20 replication forks were analyzed. (C, D) Long/short ratios were calculated for at least 20 replication forks for cell lines with indicated genotypes. Median values are indicated by the red lines; averages of the medians from three independent analyses are shown in (D). Mann-Whitney-Wilcoxon test, ns, not

significant.

Fig. S6 Dual roles of PARP1 in TDP1-mediated repair of stalled TOP1ccs and Polε exonuclease-mediated fork slowing

(A, B) Sensitivity of DT40 cells with the indicated genotypes to treatment with CPT for 48 h. The drug dose is displayed on the x-axis using a linear scale, while the cell survival percentage is displayed on the y-axis using a logarithmic scale. Data represent the mean ± standard deviation, as in Figure 1A.

Fig. S7. Polε-exonuclease and PARP1 antagonize RECQ1-mediated FR resolution

DNA fiber analysis was performed as in Figure 3A. Data are presented as in Supplementary Figure S3. Wild-type, *PARP1*$^{-/-}$, *POLE1*$^{exo-/-}$, *RECQ*$^{-/-}$, *PARP1*$^{-/-}$/ *RECQ*$^{-/-}$, and *POLE1*$^{exo-/-}$/*RECQ*$^{-/-}$ clones were examined twice independently. Medians and SEs for each test are indicated in the boxes.

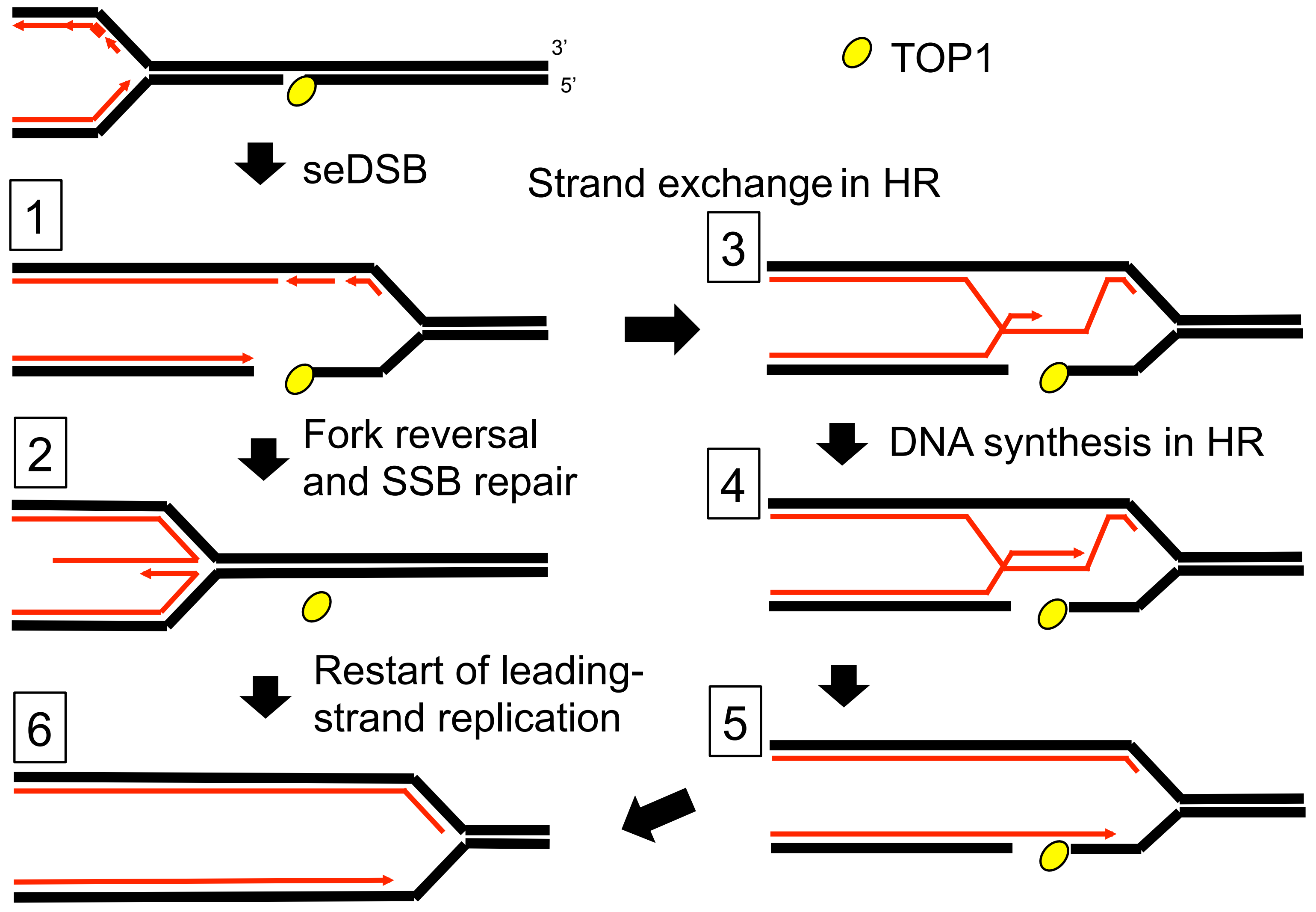
3'
5'
TOP1
seDSB
1
Strand exchange in HR
3
2
Fork reversal
and SSB repair
DNA synthesis in HR
4
Restart of leading-
strand replication
6
5

Fig. S2_1

A

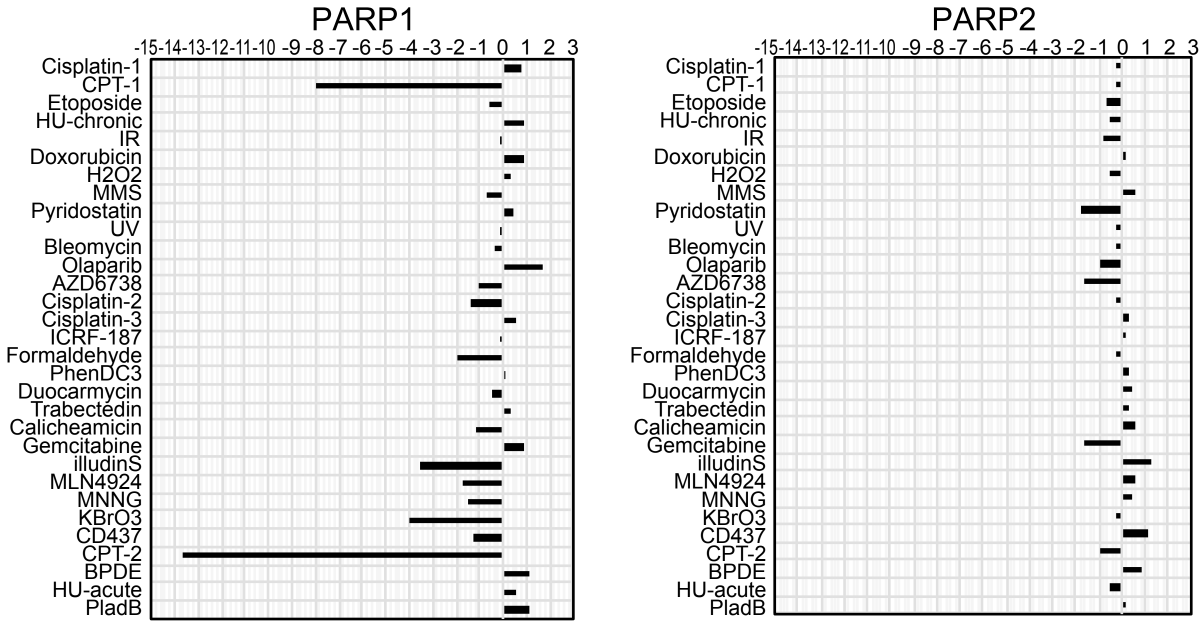
PARP1
PARP2
-15 -14 -13 -12 -11 -10 -9 -8 -7 -6 -5 -4 -3 -2 -1 0 1 2 3
Cisplatin-1
CPT-1
Etoposide
HU-chronic
IR
Doxorubicin
H2O2
MMS
Pyridostatin
UV
Bleomycin
Olaparib
AZD6738
Cisplatin-2
Cisplatin-3
ICRF-187
Formaldehyde
PhenDC3
Duocarmycin
Trabectedin
Calicheamicin
Gemcitabine
illudinS
MLN4924
MNNG
KBrO3
CD437
CPT-2
BPDE
HU-acute
PladB

Fig. S2_2

B

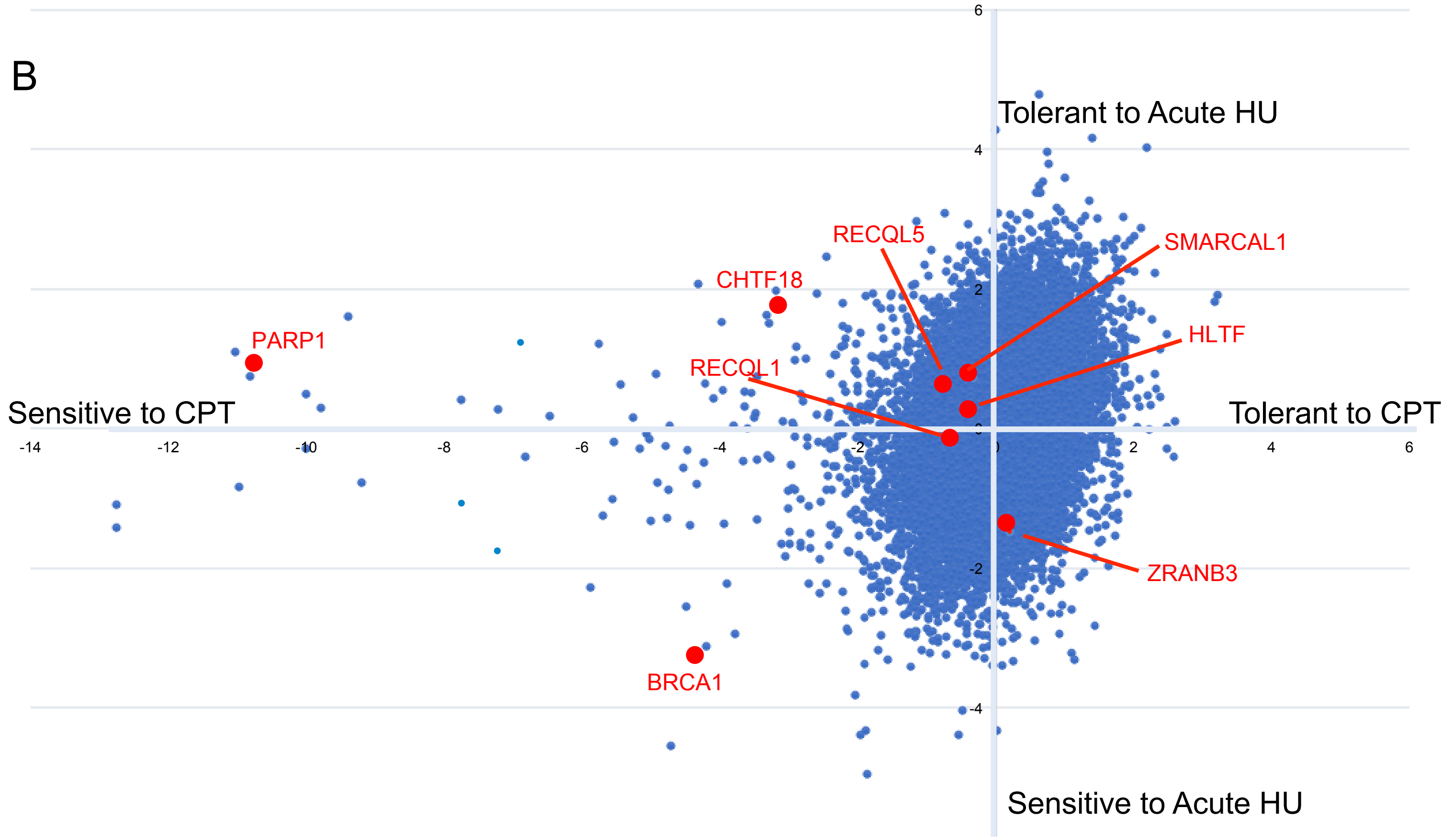

Tolerant to Acute HU
Sensitive to Acute HU
Sensitive to CPT
Tolerant to CPT
PARP1
CHTF18
RECQL5
SMARCAL1
HLTF
RECQL1
ZRANB3
BRCA1
6
4
2
-2
-4
-14
-12
-10
-8
-6
-4
2
4
6

C

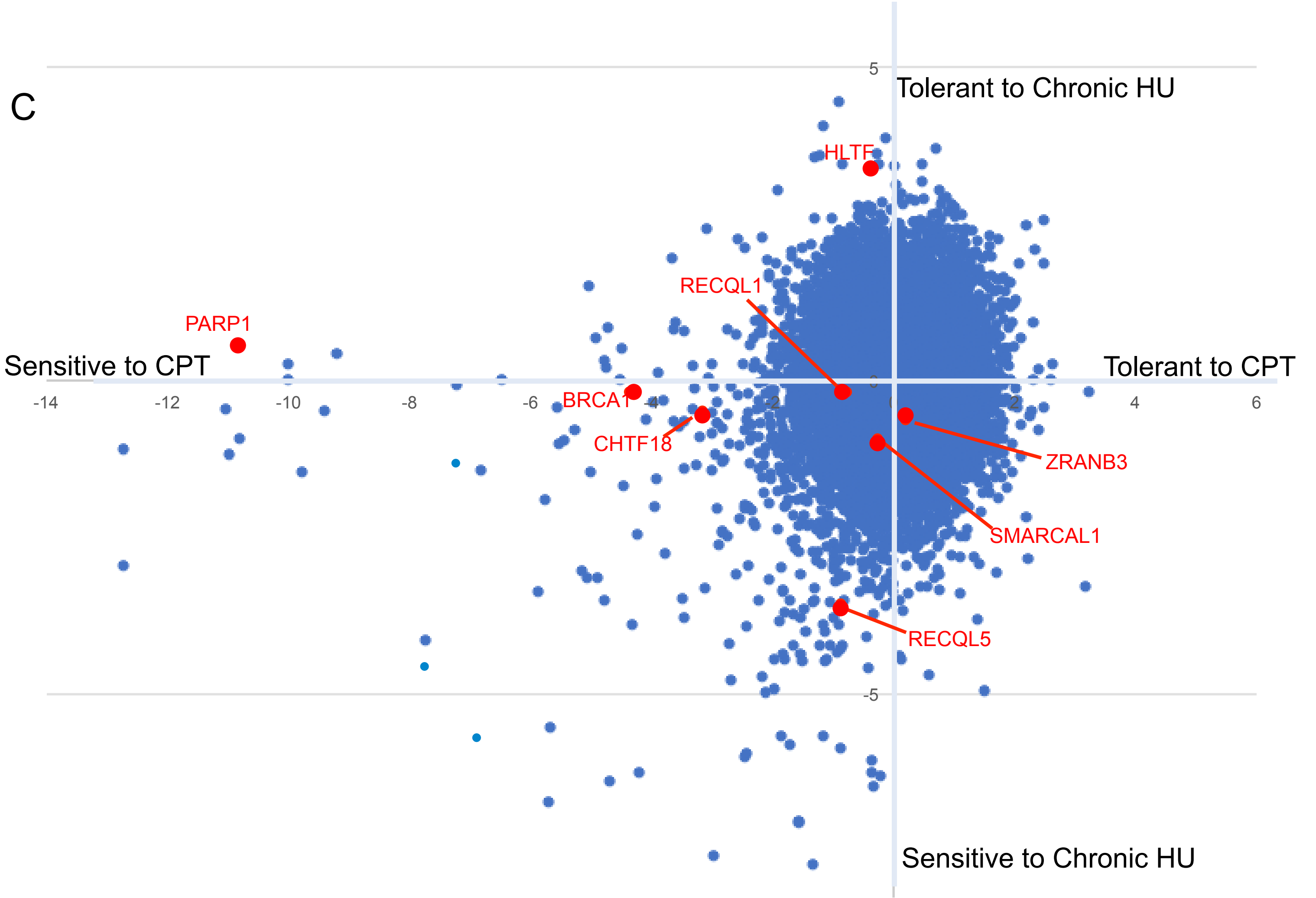

Tolerant to Chronic HU
Sensitive to Chronic HU
Sensitive to CPT
Tolerant to CPT
HLTF
RECQL1
PARP1
BRCA1
CHTF18
ZRANB3
SMARCAL1
RECQL5
5
-5
-14
-12
-10
-8
-6
4
6

Fig. S2_3

D

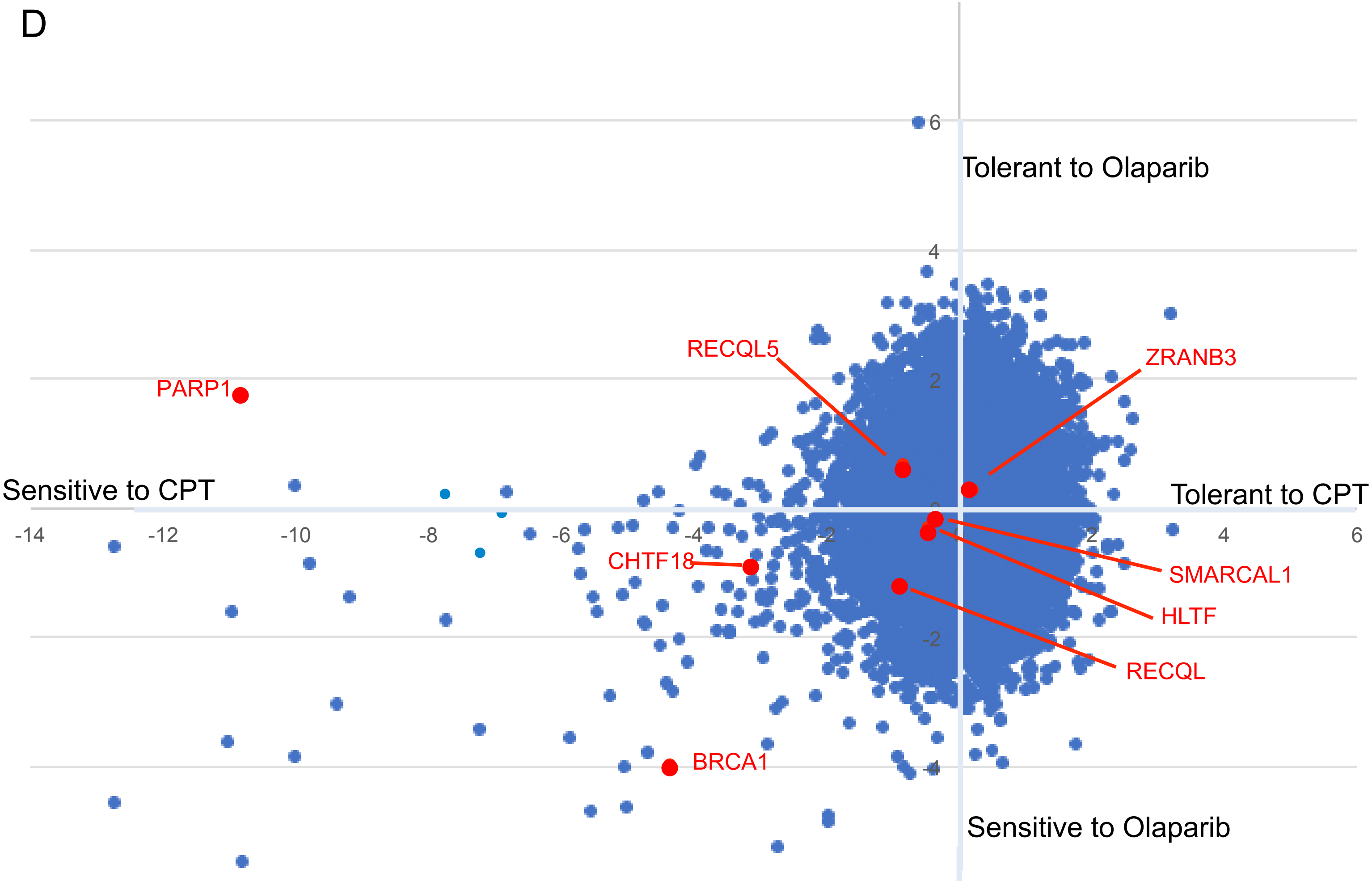

6
Tolerant to Olaparib
4
RECQL5
ZRANB3
PARP1
2
Sensitive to CPT
Tolerant to CPT
-14
-12
-10
-8
-6
-4
-2
2
4
6
CHTF18
SMARCAL1
HLTF
-2
RECQL
BRCA1
-4
Sensitive to Olaparib

E

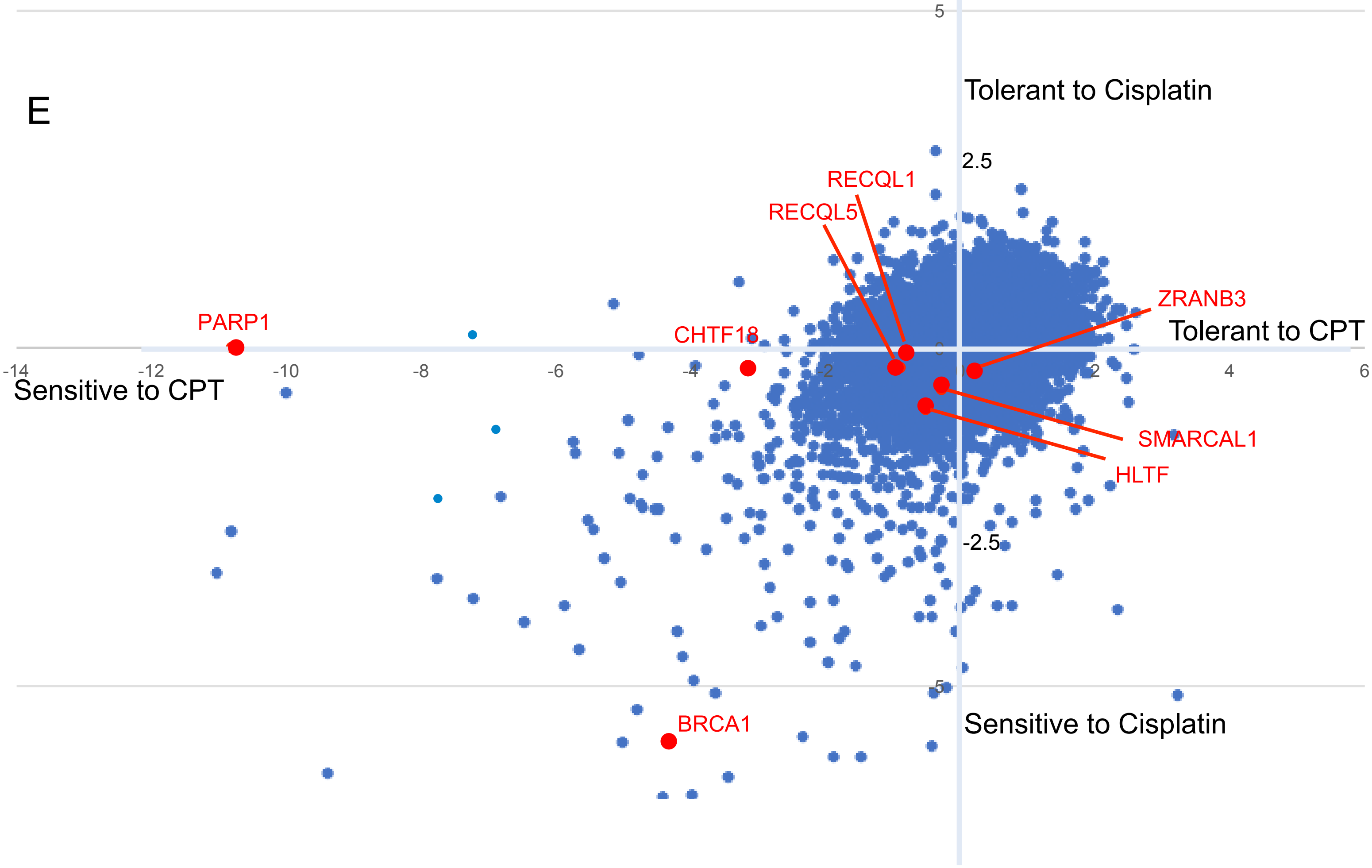

5
Tolerant to Cisplatin
2.5
RECQL1
RECQL5
ZRANB3
PARP1
CHTF18
Tolerant to CPT
-14
-12
-10
-8
-6
-4
-2
0
2
4
6
Sensitive to CPT
SMARCAL1
HLTF
-2.5
-5
BRCA1
Sensitive to Cisplatin

F

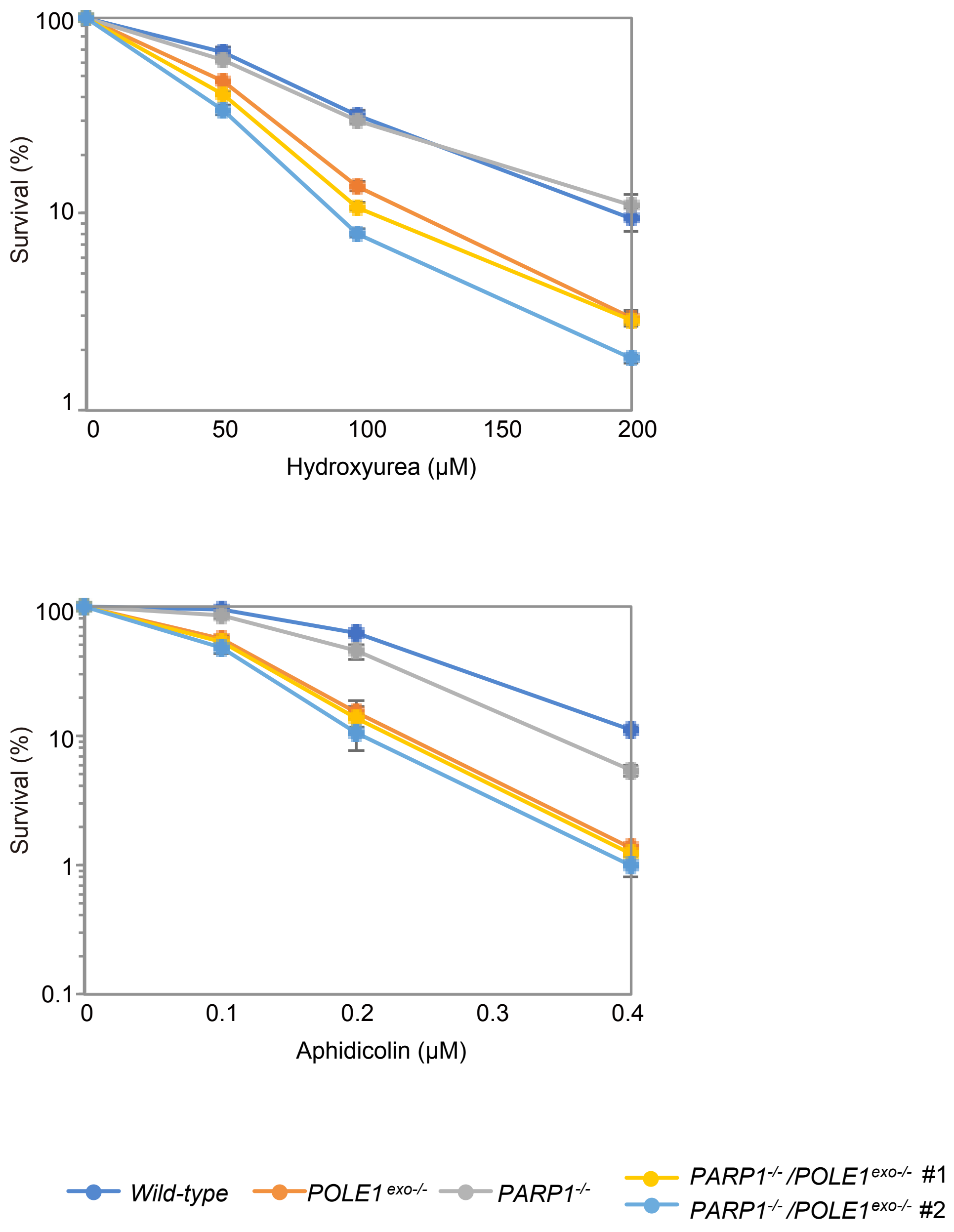
100
10
1
Survival (%)
0
50
100
150
200
Hydroxyurea (μM)
100
10
1
0.1
Survival (%)
0
0.1
0.2
0.3
0.4
Aphidicolin (μM)
Wild-type
POLE1 exo-/-
PARP1-/-
PARP1-/- /POLE1exo-/- #1
PARP1-/- /POLE1exo-/- #2

Fig. S3_1

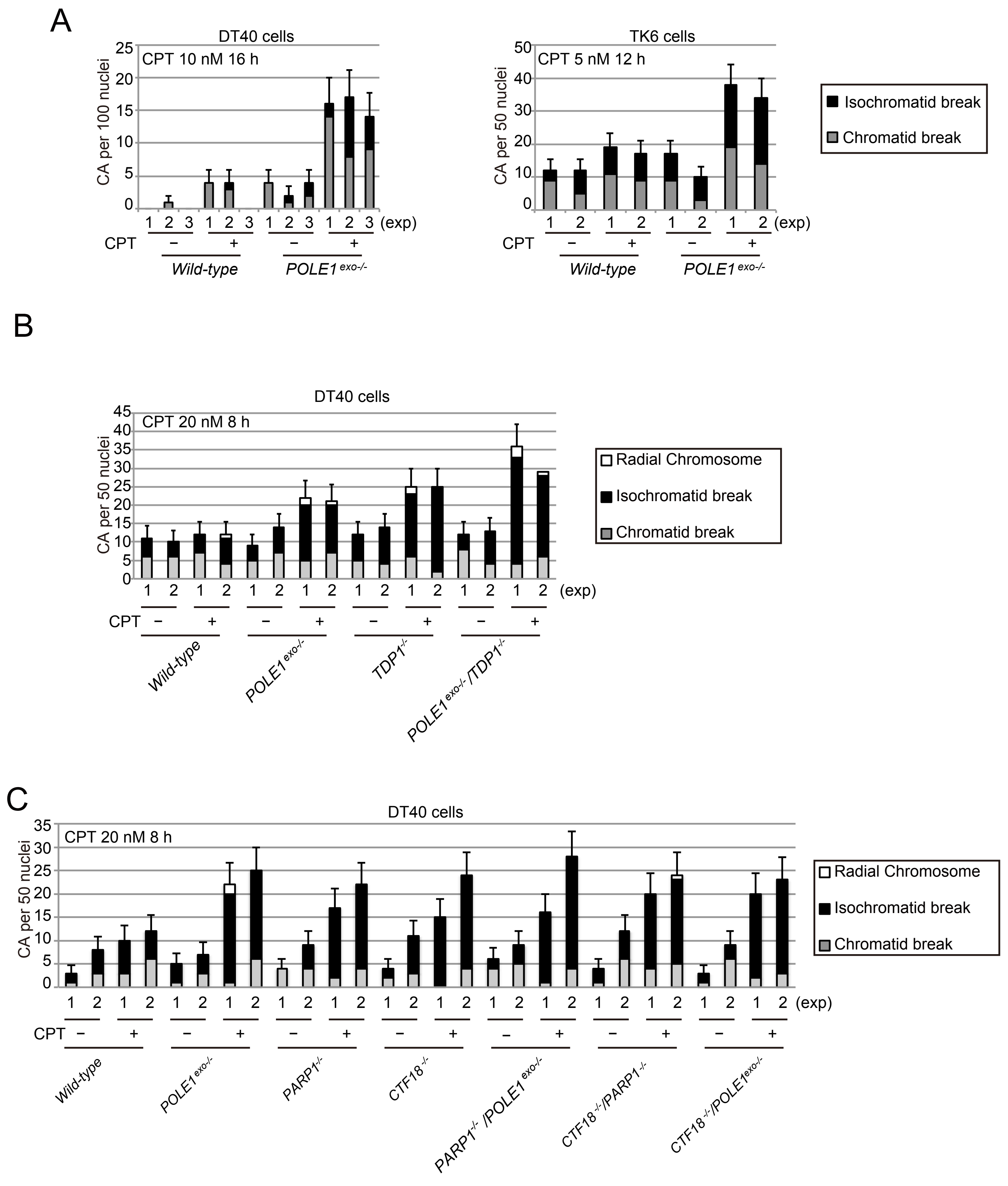
A
DT40 cells
CPT 10 nM 16 h
CA per 100 nuclei
(exp)
CPT
Wild-type
POLE1 exo-/-
TK6 cells
CPT 5 nM 12 h
CA per 50 nuclei
Isochromatid break
Chromatid break
B
DT40 cells
CPT 20 nM 8 h
CA per 50 nuclei
Radial Chromosome
Isochromatid break
Chromatid break
Wild-type
POLE1 exo-/-
TDP1-/-
POLE1 exo-/- /TDP1-/-
C
DT40 cells
CPT 20 nM 8 h
CA per 50 nuclei
Radial Chromosome
Isochromatid break
Chromatid break
Wild-type
POLE1 exo-/-
PARP1-/-
CTF18-/-
PARP1-/- /POLE1 exo-/-
CTF18 -/-/PARP1-/-
CTF18 -/-/POLE1exo-/-

Fig. S3_2

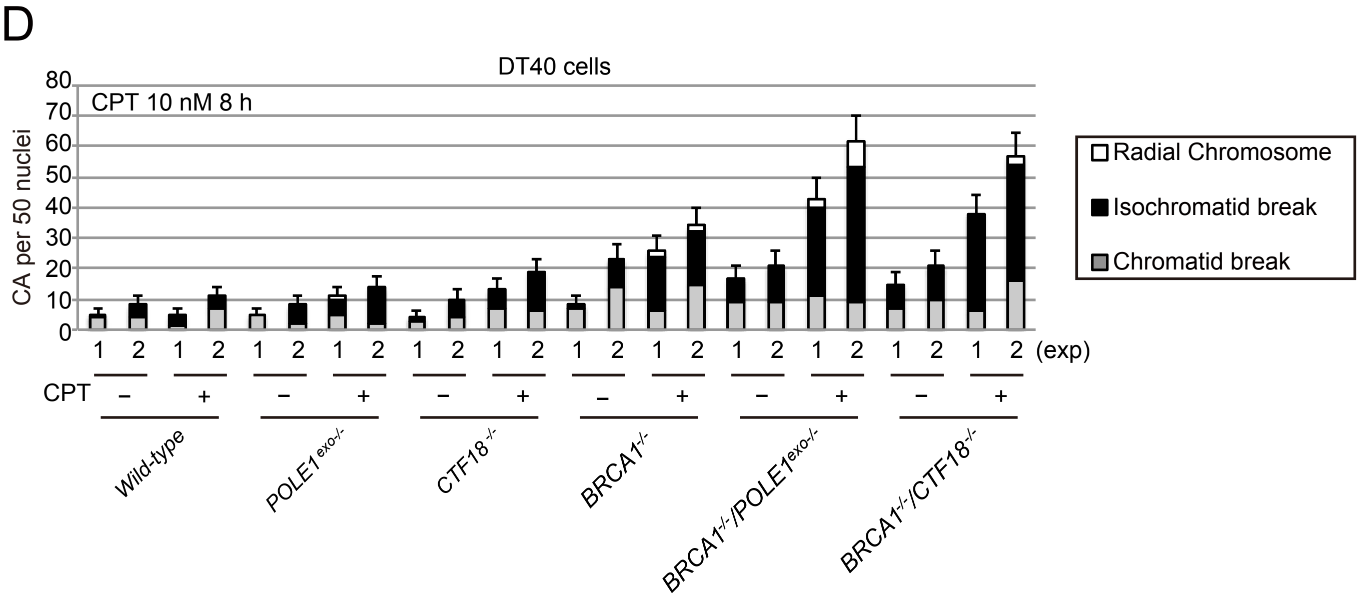
D
DT40 cells
CPT 10 nM 8 h
CA per 50 nuclei
80
70
60
50
40
30
20
10
0
Radial Chromosome
Isochromatid break
Chromatid break
1 2 1 2 1 2 1 2 1 2 1 2 1 2 1 2 1 2 1 2 1 2 1 2 (exp)
CPT – + – + – + – + – + – +
Wild-type
POLE1exo-/-
CTF18-/-
BRCA1-/-
BRCA1-/-/POLE1exo-/-
BRCA1-/-/CTF18-/-

Fig. S4_1

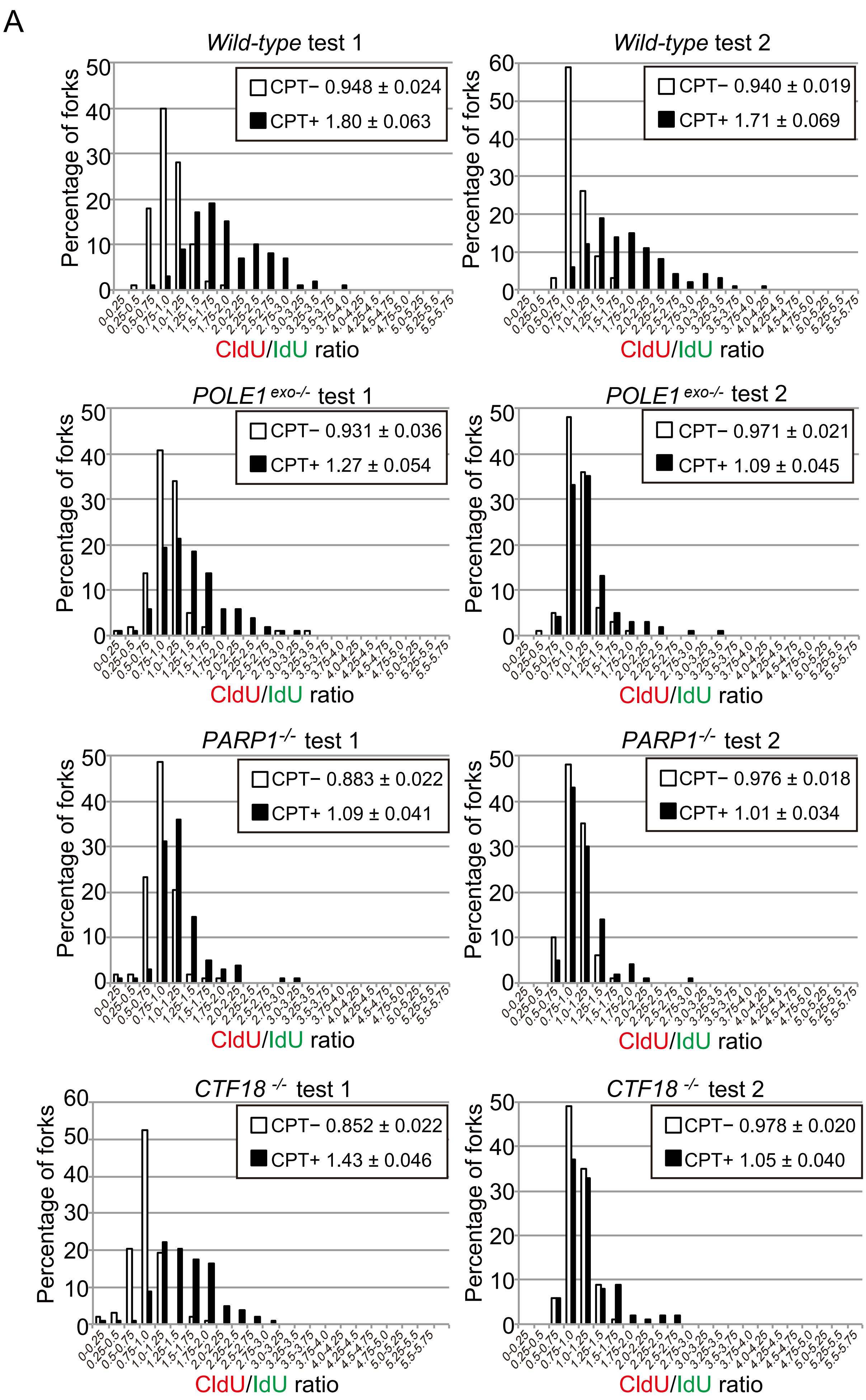
A
Wild-type test 1
CPT− 0.948 ± 0.024
CPT+ 1.80 ± 0.063
Wild-type test 2
CPT− 0.940 ± 0.019
CPT+ 1.71 ± 0.069
POLE1 exo-/- test 1
CPT− 0.931 ± 0.036
CPT+ 1.27 ± 0.054
POLE1 exo-/- test 2
CPT− 0.971 ± 0.021
CPT+ 1.09 ± 0.045
PARP1-/- test 1
CPT− 0.883 ± 0.022
CPT+ 1.09 ± 0.041
PARP1-/- test 2
CPT− 0.976 ± 0.018
CPT+ 1.01 ± 0.034
CTF18 -/- test 1
CPT− 0.852 ± 0.022
CPT+ 1.43 ± 0.046
CTF18 -/- test 2
CPT− 0.978 ± 0.020
CPT+ 1.05 ± 0.040
Percentage of forks
CldU/IdU ratio

Fig. S4_2

B

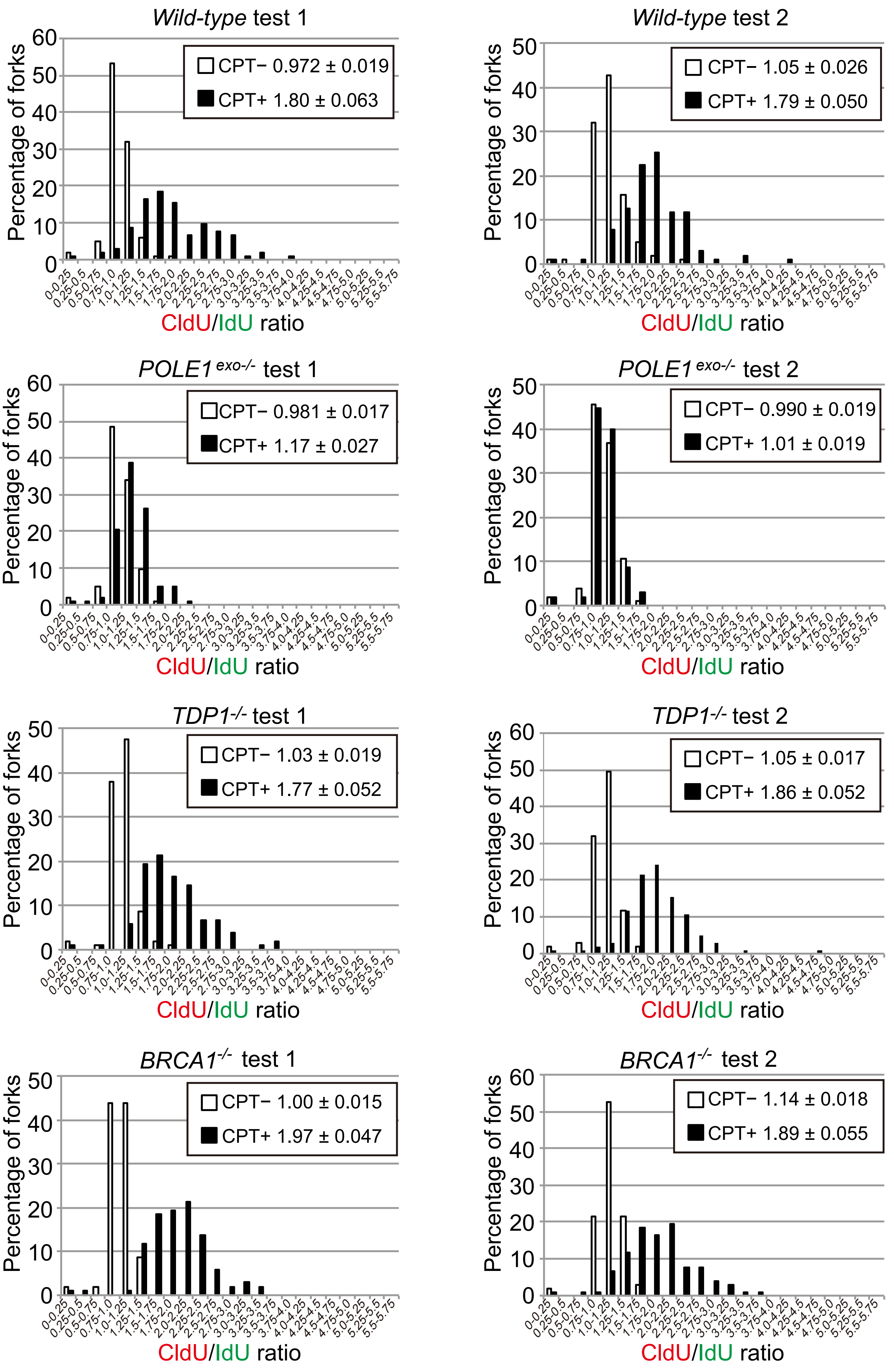

Wild-type test 1
CPT− 0.972 ± 0.019
CPT+ 1.80 ± 0.063
Wild-type test 2
CPT− 1.05 ± 0.026
CPT+ 1.79 ± 0.050
POLE1 exo-/- test 1
CPT− 0.981 ± 0.017
CPT+ 1.17 ± 0.027
POLE1 exo-/- test 2
CPT− 0.990 ± 0.019
CPT+ 1.01 ± 0.019
TDP1-/- test 1
CPT− 1.03 ± 0.019
CPT+ 1.77 ± 0.052
TDP1-/- test 2
CPT− 1.05 ± 0.017
CPT+ 1.86 ± 0.052
BRCA1-/- test 1
CPT− 1.00 ± 0.015
CPT+ 1.97 ± 0.047
BRCA1-/- test 2
CPT− 1.14 ± 0.018
CPT+ 1.89 ± 0.055
Percentage of forks
CldU/IdU ratio

Fig. S5

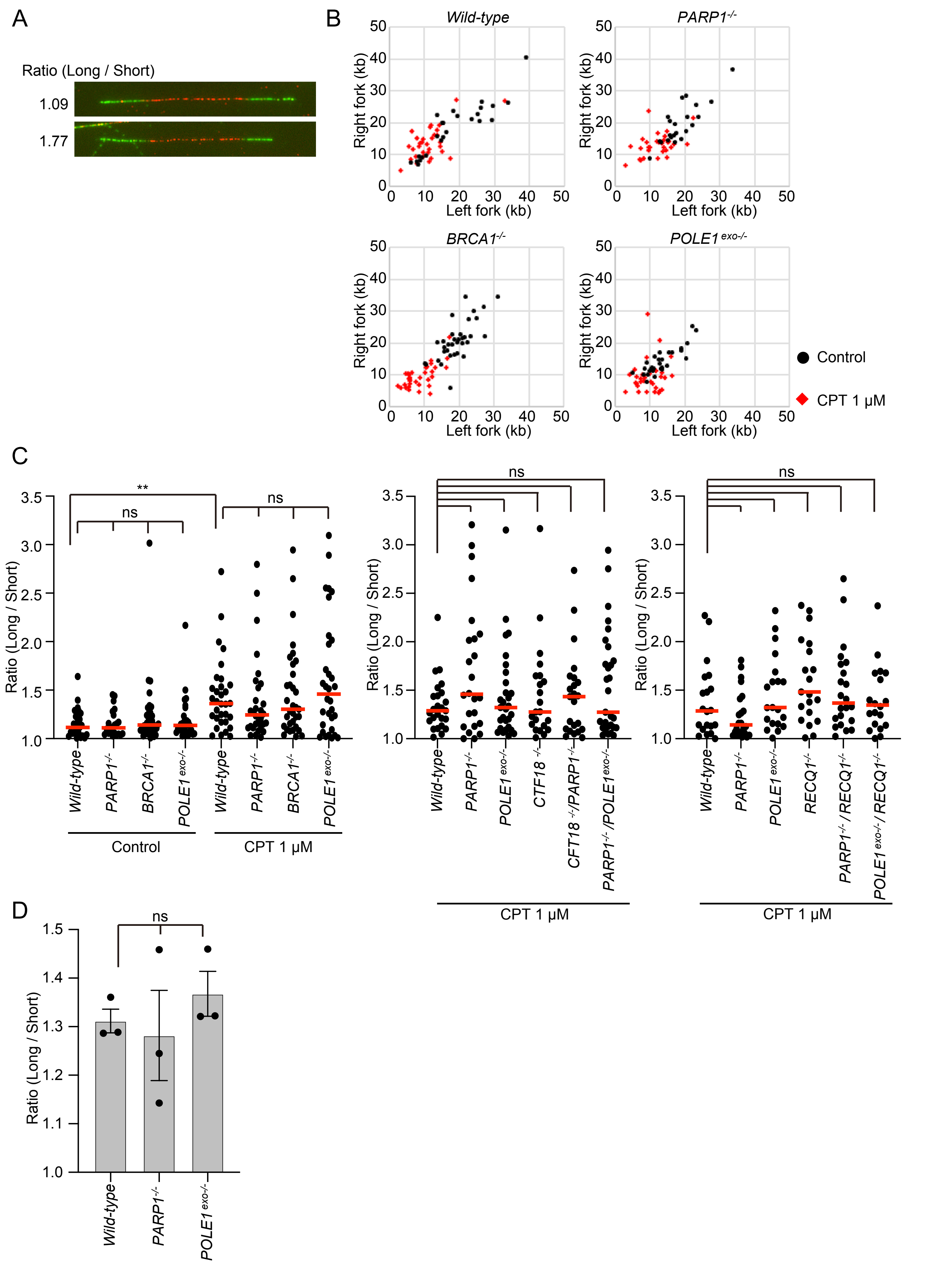
A
Ratio (Long / Short)
1.09
1.77
B
Wild-type
PARP1-/-
BRCA1-/-
POLE1 exo-/-
Right fork (kb)
Left fork (kb)
Control
CPT 1 μM
C
Ratio (Long / Short)
**
ns
Wild-type
PARP1-/-
BRCA1-/-
POLE1 exo-/-
CTF18 -/-
CFT18 -/-/PARP1-/-
PARP1-/- /POLE1 exo-/-
RECQ1-/-
PARP1-/- / RECQ1-/-
POLE1 exo-/- / RECQ1-/-
Control
CPT 1 μM
D
Ratio (Long / Short)
ns
Wild-type
PARP1-/-
POLE1 exo-/-

Fig. S6

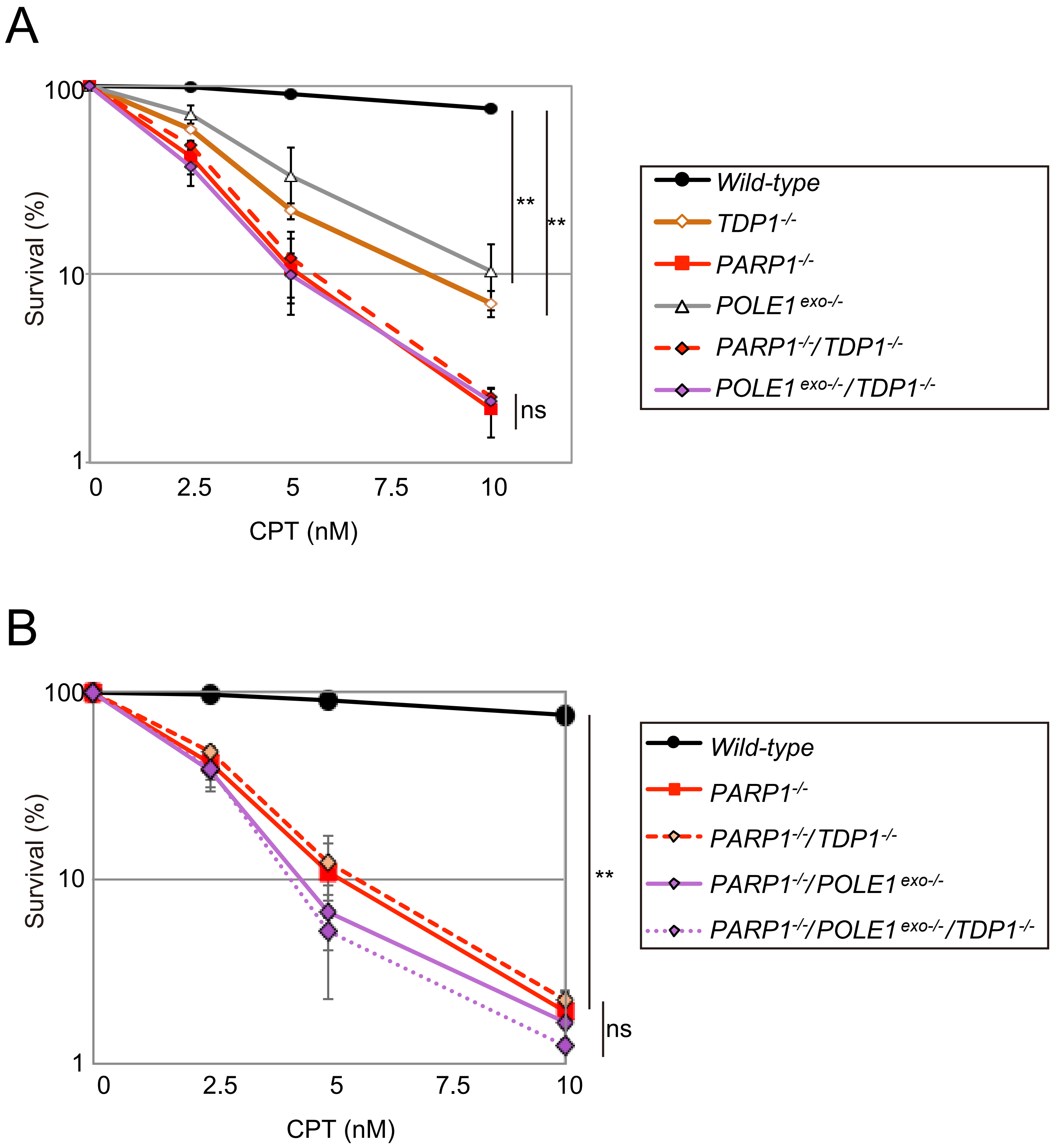

A
100
10
1
Survival (%)
0
2.5
5
7.5
10
CPT (nM)
**
**
ns
Wild-type
TDP1-/-
PARP1-/-
POLE1 exo-/-
PARP1-/-/TDP1-/-
POLE1 exo-/-/TDP1-/-
B
100
10
1
Survival (%)
0
2.5
5
7.5
10
CPT (nM)
**
ns
Wild-type
PARP1-/-
PARP1-/-/TDP1-/-
PARP1-/-/POLE1 exo-/-
PARP1-/-/POLE1 exo-/-/TDP1-/-

Fig. S7_1

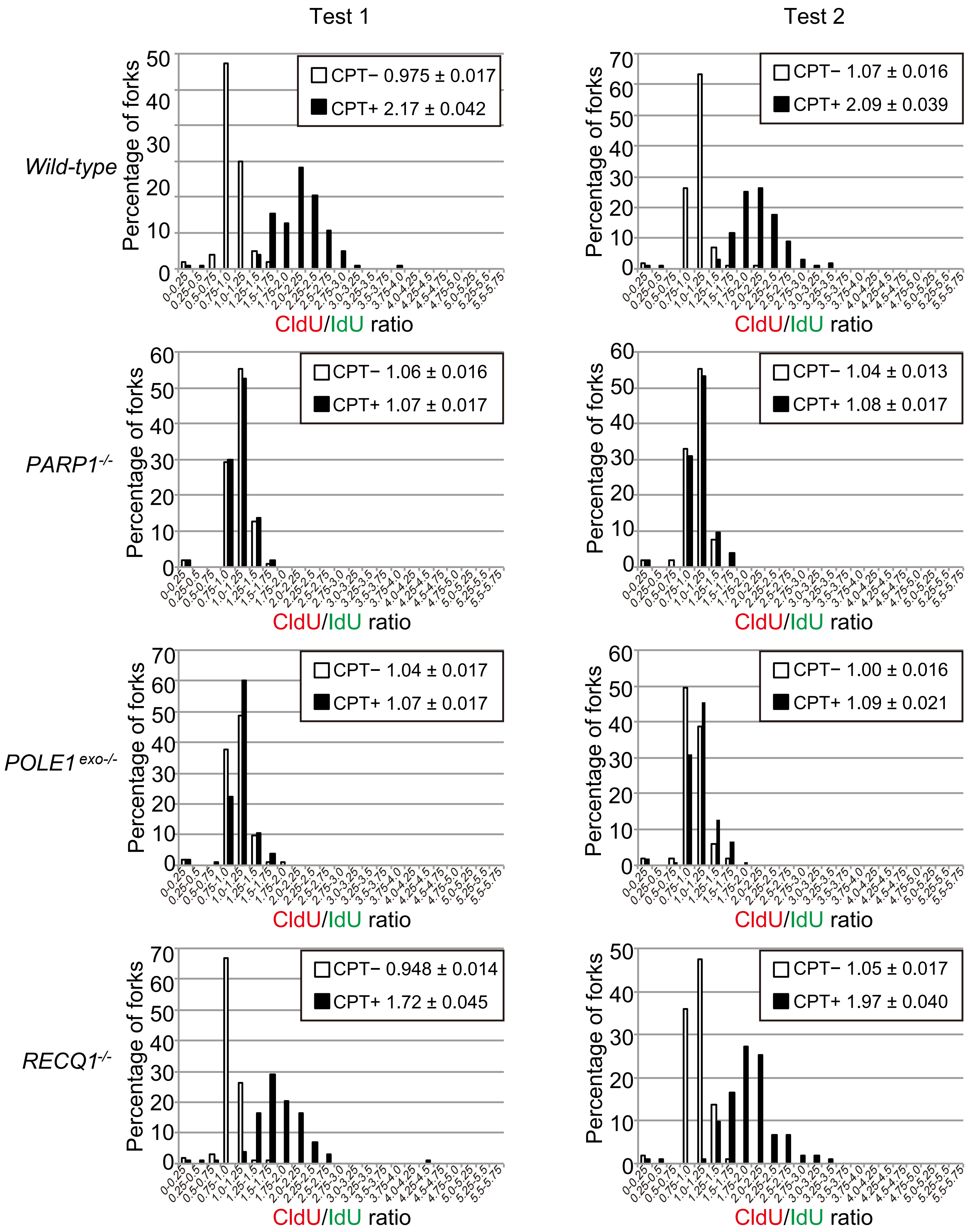

Test 1
Test 2
Wild-type
PARP1-/-
POLE1 exo-/-
RECQ1-/-
Percentage of forks
CldU/IdU ratio
CPT− 0.975 ± 0.017
CPT+ 2.17 ± 0.042
CPT− 1.07 ± 0.016
CPT+ 2.09 ± 0.039
CPT− 1.06 ± 0.016
CPT+ 1.07 ± 0.017
CPT− 1.04 ± 0.013
CPT+ 1.08 ± 0.017
CPT− 1.04 ± 0.017
CPT+ 1.07 ± 0.017
CPT− 1.00 ± 0.016
CPT+ 1.09 ± 0.021
CPT− 0.948 ± 0.014
CPT+ 1.72 ± 0.045
CPT− 1.05 ± 0.017
CPT+ 1.97 ± 0.040

Fig. S7_2

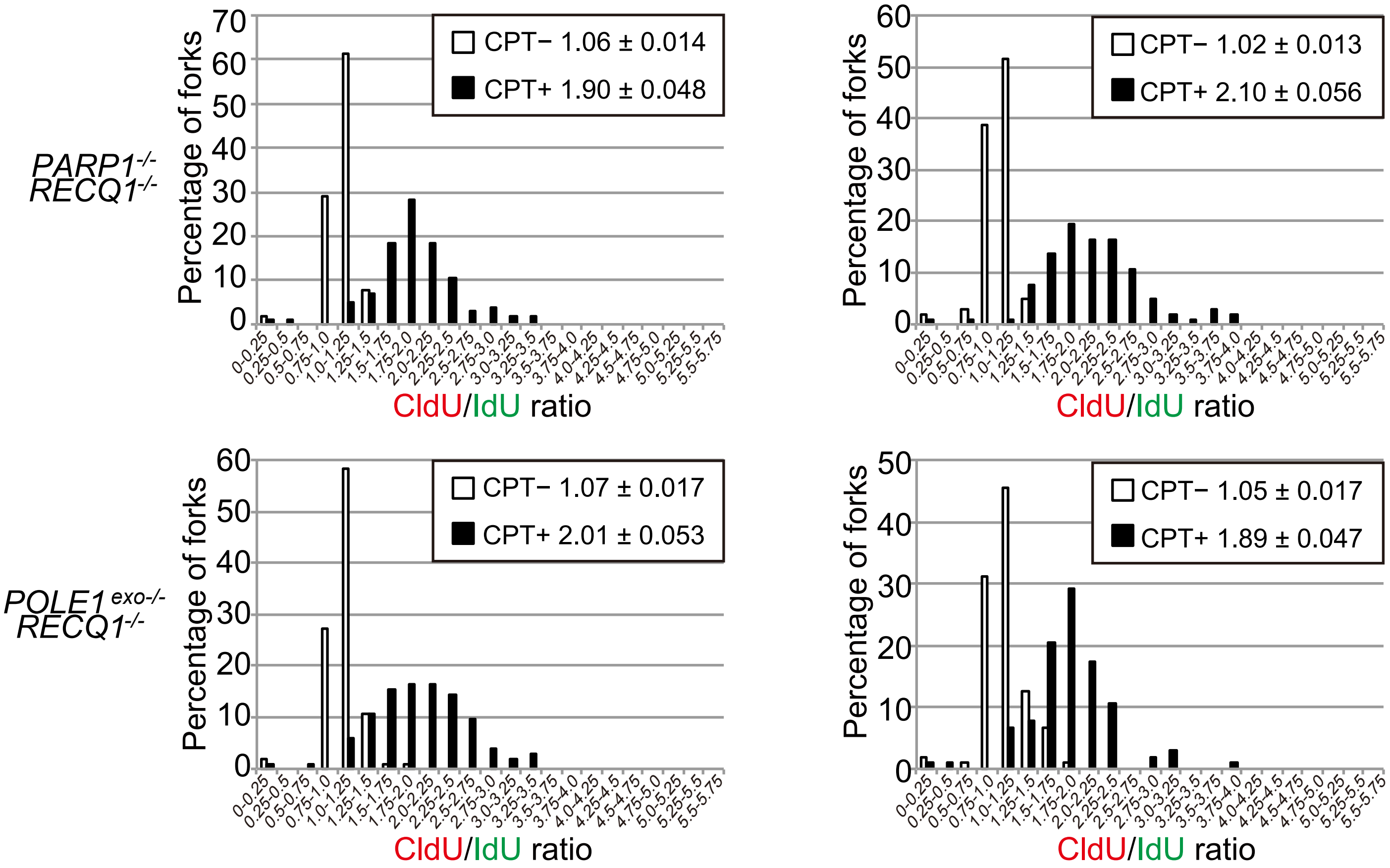
PARP1-/- RECQ1-/-
POLE1 exo-/- RECQ1-/-
Percentage of forks
CldU/IdU ratio
CPT− 1.06 ± 0.014
CPT+ 1.90 ± 0.048
CPT− 1.02 ± 0.013
CPT+ 2.10 ± 0.056
CPT− 1.07 ± 0.017
CPT+ 2.01 ± 0.053
CPT− 1.05 ± 0.017
CPT+ 1.89 ± 0.047

Supplementary Table S1

DT40 and TK6 strains used in this study

DT40

Genotype	Marker	References
Wild-type	-	48 Buerstedde
PARP1$^{-/-}$	his bsr	51 Hochegger, H
POLE1$^{exo-/-}$	bsr	37 Tsuda, M
PARP1$^{-/-}$/ POLE1$^{exo-/-}$	bsr puro his	This study
TDP1$^{-/-}$	hyg pur	29 Murai, J
POLE1$^{exo-/-}$/ TDP1$^{-/-}$	htg pur bsr	This study
PARP1$^{-/-}$/ TDP1$^{-/-}$	hyg pur eco hi	This study
PARP1$^{-/-}$/ POLE1$^{exo-/-/}$ TDP1$^{-/-}$	hyg pur eco his bsr	This study
CTF18$^{-/-}$	bsr	53 Kawasumi
CTF18$^{-/-}$/ PARP1$^{-/-}$	his bsr pur	This study
CTF18$^{-/-}$/ POLE1$^{exo-/-}$	his bsr	This study
BRCA1$^{-/-}$	pur his	52 Martin
BRCA1$^{-/-}$/ POLE1$^{exo-/-}$	bsr puro hyg	This study
BRCA1$^{-/-}$/ CTF18$^{-/-}$	bsr puro hyg	This study
RECQ1$^{-/-}$	puro bleo	This study
POLE1$^{exo-/-}$ / RECQ1$^{-/-}$	bsr puro bleo	This study
PARP1$^{-/-}$ / RECQ1$^{-/-}$	his bsr puro bleo	This study

TK6

Genotype	Marker	References
Wild-type	-	49 Levy, J.A
PARP1$^{-/-}$	his pur	50 Demin, A.A
POLE1$^{exo-/-}$	neo	37 Tsuda, M
PARP1$^{-/-}$/ POLE1$^{exo-/-}$	his pur neo	This study
POLE1$^{exo-/-}$/AAVS1::ef-1α-POLE1 clone #1	neo bsr	This study
POLE1$^{exo-/-}$/AAVS1::ef-1α-POLE1	neo bsr	This study

clone #2		
PARP1$^{-/-}$/ *POLE1*$^{exo-/-}$/*AAVS1::ef-1α-POLE1* clone #1	his pur neo bsr	This study
PARP1$^{-/-}$/ *POLE1*$^{exo-/-}$/*AAVS1::ef-1α-POLE1* clone #2	his pur neo bsr	This study
